# A new nano-engineered hierarchical membrane for concurrent removal of surfactant and oil from oil-in-water nanoemulsion

**DOI:** 10.1038/srep24365

**Published:** 2016-04-18

**Authors:** Detao Qin, Zhaoyang Liu, Hongwei Bai, Darren Delai Sun, Xiaoxiao Song

**Affiliations:** 1 Energy Research Institute @ NTU, Interdisciplinary Graduate School, Nanyang Technological University, 639798, Singapore; 2 Qatar Environment and Energy Research Institute (QEERI), Hamad bin Khalifa University (HBKU), Qatar Foundation, PO Box 5825, Doha, Qatar; 3 Energy Research Institute @ NTU, Nanyang Technological University, 639798, Singapore; 4 School of Civil and Environmental Engineering, Nanyang Technological University, 639798, Singapore

## Abstract

Surfactant stabilized oil-in-water nanoemulsions pose a severe threat to both the environment and human health. Recent development of membrane filtration technology has enabled efficient oil removal from oil/water nanoemulsion, however, the concurrent removal of surfactant and oil remains unsolved because the existing filtration membranes still suffer from low surfactant removal rate and serious surfactant-induced fouling issue. In this study, to realize the concurrent removal of surfactant and oil from nanoemulsion, a novel hierarchically-structured membrane is designed with a nanostructured selective layer on top of a microstructured support layer. The physical and chemical properties of the overall membrane, including wettability, surface roughness, electric charge, thickness and structures, are delicately tailored through a nano-engineered fabrication process, that is, graphene oxide (GO) nanosheet assisted phase inversion coupled with surface functionalization. Compared with the membrane fabricated by conventional phase inversion, this novel membrane has four times higher water flux, significantly higher rejections of both oil (~99.9%) and surfactant (as high as 93.5%), and two thirds lower fouling ratio when treating surfactant stabilized oil-in-water nanoemulsion. Due to its excellent performances and facile fabrication process, this nano-engineered membrane is expected to have wide practical applications in the oil/water separation fields of environmental protection and water purification.

Surfactant-stabilized oil/water nanoemulsions (typical droplet size: 20~200 nm) are widely encountered in various industries including oil refinery, pharmaceutical, cosmetics, food, *etc*^1^,^2^,^3^,^4^,^5^,^6^,^7^,^8^,^9^. To kinetically stabilize nanoemulsion, surfactant is added with considerable amount in oil/water nanoemulsions^10^,^11^,^12^. These oil/water nanoemulsions have aroused growing environmental and health concerns because of (1) the persistent stability and high transportability of nanosized oil droplet in environment^13^,^14^,^15^,^16^, and (2) the severe eco-toxicity of surfactant due to its strong bio-reactivity^17^,^18^,^19^.

The complete separation of oil/water nanoemulsion remains an extreme challenge due to the co-existence of nanometer-sized oil droplets and tiny surfactant molecules (typical surfactant micelle radius: <10 nm, Table S1)^13^,^20^. Traditional technologies, such as gravity separation, air flotation, skimming, centrifuge, *etc*. are incapable of removing emulsified oil and surfactant from nanoemulsion^21^,^22^. Membrane technology, which functions primarily on the principle of size exclusion^23^, is the most promising technology to address the challenge of complete separation of nanoemulsion. Recently, membrane development has significantly advanced in terms of separating oil from nanoemulsion^24^,^25^,^26^,^27^. On one hand, the study from Solomon *et al*. employed a hydrophobic membrane to separate water-in-oil nanoemulsion, by allowing oil to pass through membrane while repelling water^24^. But there exist intrinsic problems for hydrophobic membrane: (1) low flux subject to the high permeate (oil) viscosity, according to Hagen-Poiseuille equation^28^, and (2) severe oil-fouling resulted from the hydrophobicity of membrane material^29^,^30^. On the other hand, some studies employed a hydrophilic membrane to separate oil-in-water nanoemulsion, by allowing water to pass through membrane while repelling oil^25^,^26^,^27^. And by tailoring membrane thickness as thin as 50~220 nm, these thin hydrophilic membranes demonstrated superhigh water flux. However, the ultrathin thickness inevitably compromises the mechanical strength of these membranes, which adversely affect their practical applicability. Moreover, all the published studies have not yet addressed the crucial challenge of removing surfactant from nanoemulsion. Compared with oil removal, surfactant removal from nanoemulsion by membrane is much more challenging and complicated due to: (1) tiny molecular size of surfactant, which results in low removal rate; and (2) surfactant adsorption, which leads to membrane fouling and flux decline^31^,^32^.

In this study, to tackle the challenge of concurrent removal of surfactant and oil from nanoemulsion, a novel hierarchically-structured polymeric membrane is designed and fabricated through a nano-engineered fabrication process, that is, GO assisted phase inversion process coupled with surface functionalization. This novel membrane consists of an ultrathin nanostructured selective layer on top of a microstructured support layer. The thickness and pore structure of each layer are delicately tailored through GO assisted phase inversion technique on account of the great potentiality of GO nanosheet to engineer solution-based functional materials^33^,^34^,^35^,^36^. Following this, the topography, wettability, and electric charge of selective layer are further fine-tuned by an electroneutral hydrogel macromolecule (polyvinyl alcohol). As a result, this nano-engineered membrane possesses a nanostructured selective layer with the integrated advantages including ultrathin thickness, smooth topography, high underwater oleophobicity and electro-neutrality, which can endow itself with the properties of high water permeability, high rejections of both surfactant and oil, and low membrane fouling. And owing to the optimized structure parameters, the microstructured support layer can provide mechanical support while ensuring high water flux. Compared with the membrane fabricated by conventional phase inversion process, this novel membrane can separate surfactant stabilized oil-in-water nanoemulsion with four times higher water flux, significantly higher rejections of both oil (~99.9%) and surfactant (as high as 93.5%), and two thirds lower fouling ratio. Its good mechanical flexibility, facile fabrication process, and the capability of concurrently removing surfactant and oil from nanoemulsion endorse this new membrane with great potential for practical applications.

## Results

### Design of nanoemulsion separating membrane

Here, a hierarchically-structured membrane is elaborately designed to separate surfactant-stabilized oil/water nanoemulsion with low fouling, high flux and high selectivity. This membrane is composed of an ultrathin nanoporous selective layer on top of a microporous support layer. The selective layer is responsible for concurrently rejecting surfactant as well as oil and resisting membrane fouling; while the support layer is designated to provide robust mechanical strength with small flux-resistance. For designing such hierarchical membrane, the permeability, selectivity (rejection), and antifouling capability are the most important properties to be considered. Generally, the pure water permeability (PWP) of this hierarchical membrane is derived as equation 1 according to Hagen-Poiseuille law^28^. The rejection of solid solutes by this membrane can be modeled based upon size exclusion principle using Ferry equation (equation 2)^37^. And according to resistances-in-series model^38^, the theoretically maximum water flux of hierarchical membrane during nanoemulsion separation process can be derived as equation 3, with the transmembrane pressure (TMP) set equal to breakthrough pressure^39^.


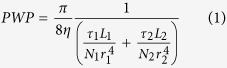


where *η* is the viscosity of permeate; *τ*_*1*_ is selective layer tortuosity, *L*_*1*_ is selective layer thickness, *r*_*1*_is selective layer pore radius, *N*_*1*_ is pore number density of selective layer; *τ*_*2*_ is support layer tortuosity, *L*_*2*_ is support layer thickness, *r*_*2*_is support layer pore radius, *N*_*2*_ is pore number density of support layer.


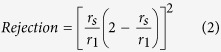


where *r*_*s*_ is solute radius. And this equation is applicable when *r*_*1*_ ≥ *r*_*s*_ ≥ 0.


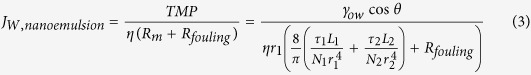


where *J*_*W,nanoemulsion*_ is the water flux with nanoemulsion as feed solution, *R*_*m*_ is the resistance induced by membrane itself, *R*_*fouling*_ is fouling induced resistance, *γ*_*ow*_ is oil-water interfacial tension, *θ* is oil contact angle of membrane surface in the presence of water. The elaborate mathematical analyses are provided in Supplementary Information (SI).

As demonstrated in the above formulae, the pore radius of selective layer (*r*_*1*_) plays an important but complicated role in membrane separation of nanoemulsion. Hierarchical membrane with larger *r*_*1*_ has higher water permeability. However, larger *r*_*1*_ leads to poorer rejection and may cause severer fouling (*R*_*fouling*_ can be regarded as a function of *r*_*1*_). In nanoemulsion, oil droplets at submicrometer scale and surfactant in the size as small as several nanometers are the two solutes to be rejected. Meanwhile, they are also foulants that can significantly degrade membrane separation performance. Therefore, the pore radius of membrane selective layer should be designed at nanometer scale, which should take account of the ability to reject the smaller solute (*i.e*. surfactant). The merits by doing so include (1) attaining high removals of both surfactant and oil, and (2) preventing membrane from internal fouling otherwise surfactant or oil droplet entering membrane pores and plugging these pores (see more analyses on the role of *r*_*1*_ in SI). Because typical micelle radius of the surfactant used here (Triton X-100) is 2~12 nm (Table S1), *r*_*1*_ of our hierarchical membrane is purposely designed below 5 nm.

Noteworthily, another three membrane intrinsic properties other than *r*_*1*_ that deserve more attention are pore number density (*N*_*1*_ and *N*_*2*_), tortuosity (*τ*_*1*_ and *τ*_*2*_), and selective layer thickness (*L*_*1*_). This is because the increase of *N* or the decrease of *L*_*1*_ or *τ* can significantly enhance *J*_*W,nanoemulsion*_ without compromising membrane rejections and mechanical integrity, as indicated by equation 3. In this study, nano-engineering is explored for its potentialities to increase *N* and decrease *L*_*1*_ or *τ*.

Moreover, *J*_*W,nanoemulsion*_ is determined by both *R*_*m*_ and *R*_*fouling*_. This means only the membrane with excellent antifouling capability (small *R*_*fouling*_) can achieve constantly high water flux during nanoemulsion separation process. Therefore, the surface properties including pore structure, topography roughness, wettability, and electric charge must be finely tuned to minimize the interactions between foulants and membrane. To guarantee this, as-designed hierarchical membrane is equipped with a smooth surface because foulants are inclined to clog in the valley regions of rough topography and hence membrane with smoother surface possesses better antifouling capability^40^. Simultaneously, the membrane surface is engineered to be underwater oleophobic, which not only diminishes *R*_*fouling*_ through reducing the affinity of oily foulants on membrane surface but also elevates breakthrough pressure with bigger cos *θ* (equation 3).

### Properties of nano-engineered hierarchical membrane

**Figure 1:**
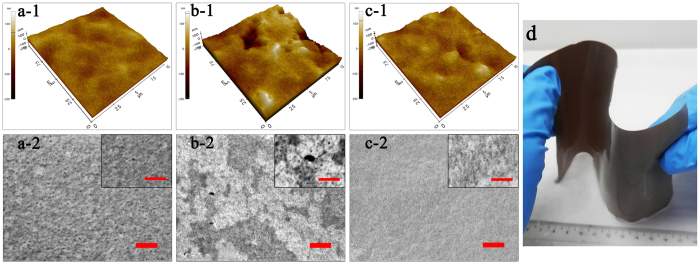
The structure of membrane selective layer. (**a**) P membrane, (**b**) GO-P membrane, (**c**) GO-P-S membrane. (**a-1**~**c-1**) AFM images of membrane topography. (**a-2**~**c-2**) FESEM images of membrane top surface, scale bar 300 nm; the inserted figures are enlarged FESEM images of membrane top surface, scale bar 150 nm. (**d**) Optical photo of GO-P-S membrane which demonstrates its outstanding mechanical flexibility.

**Figure 2:**
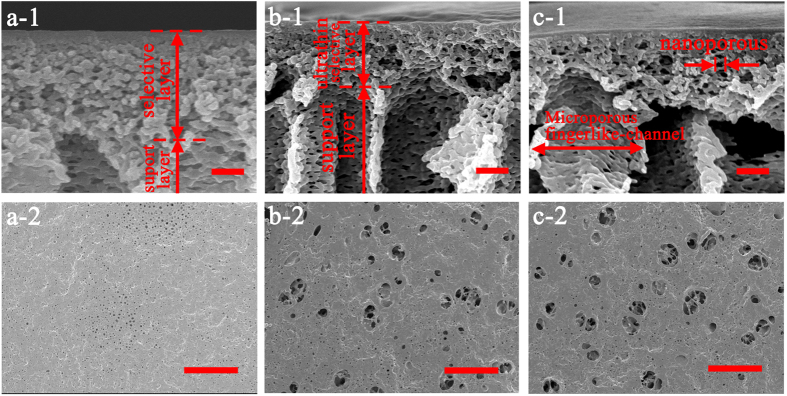
The structure of membrane cross-section and bottom surface. (**a**) P membrane, (**b**) GO-P membrane, (**c**) GO-P-S membrane. (**a-1**~**c-1**) Enlarged FESEM images of membrane cross-section, scale bar 300 nm. (**a-2**~**c-2**) FESEM images of membrane bottom surface, scale bar 10 μm.

Guided by the above design rationale, the hierarchically-structured membrane is synthesized through GO assisted phase inversion coupled with delicate surface functionalization towards the complete separation of nanoemulsion. This nano-engineered membrane is coded as “GO-P-S” membrane; while for the comparison purpose, GO assisted phase inversion constructed membrane without surface functionalization is coded as “GO-P” membrane, and conventional phase inversion constructed membrane with neither GO nano-engineering nor surface functionalization is coded as “P” membrane, respectively. Here, GO nanosheet (Fig. S3) is chosen to adjust the phase inversion process so as to tailor the structure of resultant membrane. The mechanism is the superhydrophilicity of GO nanosheet can accelerate phase inversion process through inducing a faster exchange of water intrusion with solvent extrusion^41^,^42^. As the result of GO nano-engineering, phase inversion constructed membrane is tuned to be more porous (Fig. 1a,b). Observed by FESEM image, the average pore radius of membrane selective layer (membrane top surface) *r*_*1*_ is enlarged from ~8 nm (Fig. 1a-2) to ~16 nm (Fig. 1b-2), accompanied by the emergence of a few big pores as large as 80 nm (Fig. 1b-2). More importantly, the pore number density of selective layer (*N*_*1*_) is increased remarkably from 120 μm^−2^ to 410 μm^−2^ owing to the nano-engineering by GO sheet. Simultaneously, an ultrathin nanoporous selective layer is generated as the result of GO nano-engineering, with the thickness of selective layer (*L*_*1*_) reduced by ~45% from 880 nm (Fig. 2a-1) to 490 nm (Fig. 2b-1). Meanwhile, the average pore radius of membrane bottom surface is enlarged from ~0.3 μm (Fig. 2a-2) to ~2.0 μm (Fig. 2b-2), which indicates the interconnectivity of pores in membrane cross-section gets improved (*τ* gets decreased).

However, GO assisted phase inversion alone cannot generate the perfect structure for nanoemulsion separating membrane, because a rougher membrane topography is simultaneously produced as the drawback of accelerating phase inversion process (Fig. 1a-1,b-1). Therefore, delicate surface functionalization with hydrogel macromolecule is employed to further fine-tune the structure of selective layer. As a result, the topography roughness is reduced by >1 times from 32.8 nm (Fig. 1b-1) to 14.8 nm (Fig. 1c-1). Meanwhile, this surface functionalization is able to fill those previously emerged big pores ~80 nm, reducing the pore radius of selective layer to ~5 nm (Fig. 1c-2). Although the pore number density of selective layer (*N*_*1*_) is simultaneously reduced by 33% to 275 ± 30 μm^−2^ after surface functionalization, the *N*_*1*_ value of GO-P-S membrane is still one times higher than that of P membrane (see more analysis of *N*_*1*_ on Table S2). Noteworthily, this surface functionalization does not alter the thickness of nanoporous selective layer (Fig. 2b-1,c-1) or the structure of microporous support layer (Fig. 2b-2,c-2). In addition, our nano-engineered hierarchical membrane demonstrates outstanding mechanical flexibility that can hardly be possessed by inorganic/ceramic membranes (Fig. 1d).

**Figure 3:**
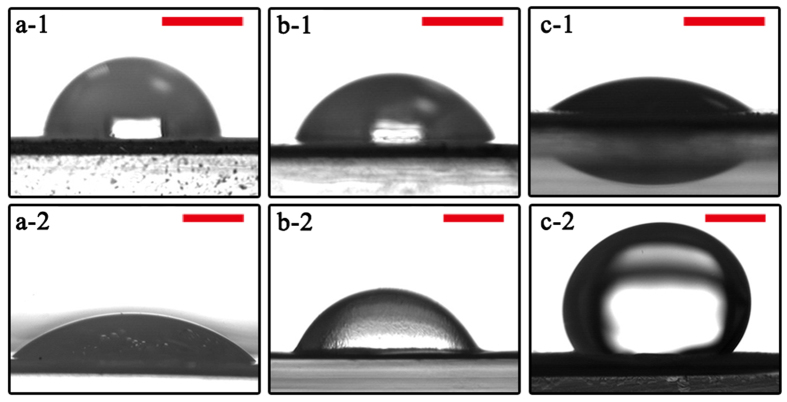
The surface wettability of membrane. (**a**) P membrane, (**b**) GO-P membrane, (**c**) GO-P-S membrane. (**a-1**~**c-1**) Water contact angles in air, scale bar, 1 mm. (**a-2**~**d-2**) Underwater oil contact angles, scale bar, 1 mm.

More importantly, this surface functionalization also generates a qualitative improvement in membrane wettability (Fig. 3). Although GO nano-engineering is able to reduce water contact angle from 84.5° ± 2.3° (Fig. 3a-1) to 64.5° ± 5.2° (Fig. 3b-1), phase inversion constructed membrane remains to be oleophilic, as evidenced by <70° underwater oil contact angle of GO-P membrane (Fig. 3b-2). In contrast, the hydrogel macromolecules immobilized on membrane surface endow GO-P-S membrane with not only high hydrophilicity but also high underwater oleophobicity, as evidenced by its water contact angle in air reduced to as low as 30.5° ± 3.3° (Fig. 3c-1) and underwater oil contact angle increased to 141.6° ± 3.5° (Fig. 3c-2).

**Figure 4:**
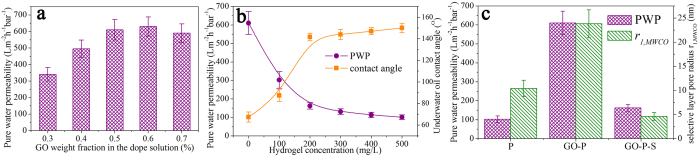
The pure water permeability and neutral solute selectivity of membrane. (**a**) The effect of GO weight fraction in nanocomposite dope solution on pure water permeability (PWP) of GO-P membrane. (**b**) The effect of hydrogel concentration on PWP and underwater oil contact angle of GO-P-S membrane. (**c**) Membrane PWP and selective layer pore radius (*r*_*1, MWCO*_, which is calculated from MWCO).

The pure water permeability (PWP) of GO-P membrane can be tuned through controlling the weight fraction of GO sheet in nanocomposite dope solution. The PWP of GO-P membrane is enhanced significantly along with the increase of GO weight fraction from 0.3 wt% to 0.5 wt% while stabilized around 620 L m^−2^ h^−1^ bar^−1^ as GO weight fraction exceeding 0.5 wt% (Fig. 4a). In this study, GO-P membrane refers to GO weight fraction of 0.5 wt% in nanocomposite dope solution. Furthermore, delicate surface functionalization can be achieved through finely adjusting the concentration of hydrogel solution, with the positive correlation between underwater oil contact angle and hydrogel concentration revealed in Fig. 4b. Particularly, the increase of underwater oil contact angle becomes relatively insignificant as hydrogel concentration exceeding 200 mg/L. Therefore, the hydrogel concentration is optimized as 200 mg/L in order to balance surface wettability and membrane permeability.

Figure 4c compares as-synthesized P, GO-P and GO-P-S membranes in PWP and neutral solute selectivity. GO nano-engineering can enhance membrane permeability by 5 times from 102 ± 18 L m^−2^ h^−1^ bar^−1^ to 610 ± 62 L m^−2^ h^−1^ bar^−1^. At the optimum hydrogel concentration, the PWP of GO-P-S membrane is tuned down to be 162 ± 18 L m^−2^ h^−1^ bar^−1^. This PWP value is still 60% higher than that of P membrane, which is ascribed to the higher selective layer pore number density (*N*_*1*_) and thinner selective layer thickness (*L*_*1*_) in GO-P-S membrane compared with P membrane. Correspondingly, the selective layer pore radius calculated from MWCO (*r*_*1,MWCO*_) is increased from 10.4 ± 1.7 nm to 23.8 ± 2.9 nm due to GO nano-engineering, and further reduced to 4.6 ± 0.8 nm due to surface functionalization. This trend is consistent with FESEM observations as aforementioned. Eventually, a hierarchical membrane with high permeability, high underwater oil repellency and smooth topography is constructed as designed by nano-engineering.

### Synthetic oil-in-water nanoemulsions

**Figure 5:**
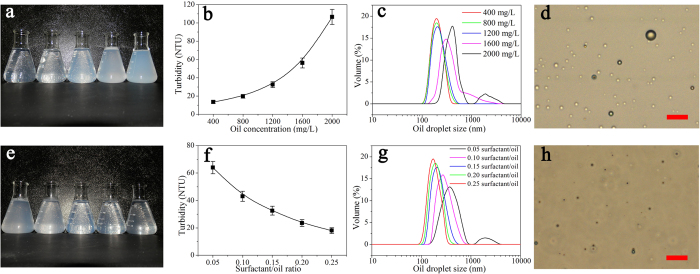
The characterizations of oil-in-water nanoemulsions. (**a**) Optical photo (from left to right, oil concentration is increased from 400 mg/L to 2000 mg/L by 400 mg/L increment for each flask), (**b**) turbidity data and (**c**) dynamic light scattering (DLS) result of nanoemulsions under different oil concentrations (The surfactant/oil ratio is kept as 0.15). (**d**) Optical microscopy image of 2000 mg/L nanoemulsion with 0.15 surfactant/oil ratio (scale bar, 2 μm). (**e**) Optical photo (from left to right, surfactant/oil ratio is increased from 0.05 to 0.25 by 0.05 increment for each flask), (**f**) turbidity data and (**g**) DLS result of nanoemulsions under different surfactant/oil ratios (The oil concentration is kept as 1200 mg/L). (**h**) Optical microscopy image of 1200 mg/L nanoemulsion with 0.15 surfactant/oil ratio (scale bar, 2 μm).

Various oil-in-water nanoemulsions were prepared as the feed solutions for membrane separation. It’s found that both oil concentration and surfactant/oil ratio play an important role in the dispersion of oil droplets in nanoemulsion. Figure 5a–c demonstrates the positive correlation between oil droplet size and oil concentration. Generally, the synthetic nanoemulsions become less transparent as oil concentration increased (Fig. 5a), which is also evidenced by the corresponding increase of turbidity (Fig. 5b). Moreover, dynamic laser light scattering (DLS) result reveals that oil droplet distribution is shifted towards larger sizes along with the increase of oil concentration. Noteworthily, the initial increase of oil concentration from 400 mg/L to 1200 mg/L merely causes a slight increase in average droplet size from 180 nm to 211 nm. While a further increase of oil concentration to 1600 mg/L and 2000 mg/L leads to the remarkable increase in average droplet size to 316 nm and to 479 nm, respectively (Fig. 5c).

Meanwhile, Fig. 5e–g displays the negative correlation between oil droplet size and surfactant/oil ratio. The synthetic nanoemulsions exhibit more evident Tyndall phenomenon as surfactant/oil ratio increased (Fig. 5e), accompanied by the decease of turbidity (Fig. 5f). Moreover, DLS result reveals that the increase of surfactant/oil ratio from 0.05 to 0.15 is effective to narrow oil droplet size distribution and reduce average droplet size from 456 nm to 208 nm. However, the further increase of surfactant/oil ratio from 0.15 to 0.25 cannot generate such remarkable change in oil droplet size distribution, leading to the average droplet size merely decreased from 208 nm to 182 nm (Fig. 5g).

### Membrane separation of nanoemulsion

**Figure 6:**
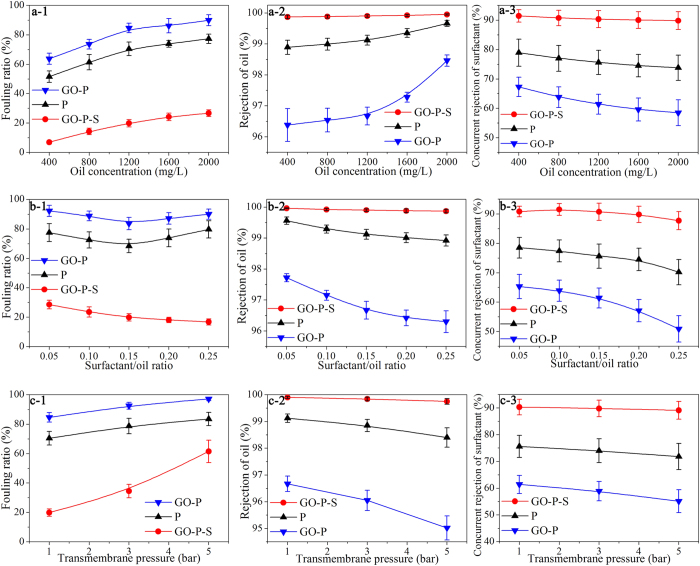
Membrane separation of oil-in-water nanoemulsion. (**a-1**) Fouling ratios, (**a-2**) oil rejections, and (**a-3**) concurrent rejections of surfactant under different oil concentrations, respectively (surfactant/oil ratio is kept as 0.15 and transmembrane pressure is 1 bar). (**b-1**) Fouling ratios, (**b-2**) oil rejections, and (**b-3**) concurrent rejections of surfactant under different surfactant/oil ratios, respectively (oil concentration is kept as 1200 mg/L and transmembrane pressure is 1 bar). (**c-1**) Fouling ratios, (**c-2**) oil rejections, (**c-3**) concurrent rejections of surfactant under different transmembrane pressures, respectively (oil concentration is 1200 mg/L and surfactant/oil ratio is 0.15).

Fouling ratio (see the definition in Methods section), oil rejection, and concurrent surfactant rejection were systematically investigated for P, GO-P and GO-P-S membranes when separating oil-in-water nanoemulsion. Figure 6a-1 shows that membrane fouling is aggravated as oil concentration increased. More importantly, GO-P-S membrane demonstrates much better antifouling capability compared with the other two membranes. For example, the fouling ratio of P membrane is increased from 51.5% to 77.3% along with the increase of oil concentration from 400 mg/L to 2000 mg/L, while the fouling ratio of GO-P-S membrane is only increased from 7.0% to 26.7% correspondingly. This means the *J*_*W,nanoemulsion*_ of GO-P-S membrane (119 L m^−2^ h^−1^) is five times as high as that of P membrane (23.2 L m^−2^ h^−1^) at 2000 mg/L oil concentration.

Figure 6a-2 shows that GO-P-S membrane outperforms the other two membranes in the rejection of oil droplets. Under the same testing conditions, the increase of oil concentration from 400 mg/L to 2000 mg/L leads to the different outcomes in oil rejection for the three membranes: (1) from 97.3% to 99.0% for P membrane, (2) from 96.4% to 98.5% for GO-P membrane, and (3) from 99.87% to 99.95% for GO-P-S membrane, respectively. The positive correlation between solute concentration and rejection observed here is quite unusual for pore-flow membrane process. On the contrary, a negative correlation between solute concentration and rejection was often reported when using pore-flow membrane to reject solid solutes, including both charged species (*e.g*. arsenic, Na_2_SO_4_) and uncharged particles (*e.g*. PEG)^43^,^44^,^45^,^46^. The sizes of those solid solutes are relatively independent on solute concentration. Differently, in nanoemulsion, oil droplet distribution can be shifted towards larger sizes along with the increase of oil concentration. Consequently, the percentage of oil droplets that exceed membrane pores in size is increased, and thus oil rejection by membrane gets enhanced.

Moreover, GO-P-S membrane demonstrates 89 ~ 93% concurrent rejection of surfactant, which is considerably higher than the other two membranes (Fig. 6a-3). Interestingly, one recent study on the separation of oil-in-water nanoemulsion reports that its membrane can reject oil at high efficiency but allows surfactant to permeate through^26^. Here, our GO-P-S membrane demonstrates a remarkable improvement in the complete removal of pollutants from nanoemulsion, for GO-P-S membrane is able to achieve high rejections of both surfactant and oil. This is probably because the pore radius of the reported membrane is in tens of nanometer while the pore radius of our GO-P-S membrane is purposely tailored to ~5 nm in order to effectively reject surfactant molecules (compared in FESEM images).

The role of surfactant needs to be carefully examined because it can influence oil droplet size distribution to a great extent. As shown in Fig. 6b-1, GO-P-S membrane demonstrates a different response to fouling from the other two membranes when surfactant/oil ratio is increased from 0.05 to 0.25 (oil concentration is kept as 1200 mg/L). For P membrane, the initial increase of surfactant/oil ratio from 0.05 to 0.15 leads to the decrease of fouling ratio from 77.5% to 68.4%, while the further increase of surfactant/oil ratio results in the bounce of fouling ratio back to 79.7%. The similar trend is also observed for GO-P membrane. Based upon the previous analyses on oil droplet size distribution, surfactant is speculated to have at least two opposite effects on membrane fouling during nanoemulsion separation process. The initial increase of surfactant concentration (0.05~0.15 surfactant/oil ratio) is effective to narrow down oil droplet size distribution, which indicates surfactant molecules exist primarily in the form of being bonded with oil to disperse it into smaller droplets. And because smaller oil droplet has weaker attraction with membrane surface, membrane fouling is mitigated within this range. However, no remarkable change in oil droplet size distribution is generated as surfactant concentration further increased (0.15~0.25 surfactant/oil ratio). This indicates there exist some surfactant molecules not bonded with oil at excessively high surfactant concentration. These surfactant molecules can undergo self-aggregation to form macromolecular assemblies^47^,^48^, and induce membrane fouling through surface adsorption, clogging valley regions of rough topography, or plugging membrane pores^31^,^49^,^50^. In such way, the excessive increase of surfactant concentration results in a counter-productive effect on fouling mitigation for P and GO-P membranes. On the contrary, GO-P-S membrane demonstrates a monotonous decrease of fouling ratio from 28.6% to 16.8% along with the increase of surfactant/oil from 0.05 to 0.25. This indicates GO-P-S membrane is able to resist not only oil-fouling but also surfactant induced fouling, which is attributed to the synergistic effect of its smooth topography and underwater oil-repellent property.

Figure 6b-2 shows that GO-P-S membrane maintains the highest rejection of oil around 99.90% under different surfactant/oil ratios. In contrast, the oil rejections of P and GO-P membranes are decreased along with the increase of surfactant/oil ratio, with the decrease rate become smaller as surfactant/oil ratio >0.15. For example, the oil rejection of GO-P membrane undergoes a remarkable decrease from 97.7% to 96.7% as surfactant/oil increased from 0.05 to 0.15, and turns to be stabilized approaching 96.3% as surfactant/oil ratio exceeding 0.15. The evident decreases in oil rejection for P and GO-P membranes as surfactant/oil ratio increased from 0.05 to 0.15 are resulted from the remarkable down-shift of oil droplet size distribution within this range.

Figure 6b-3 demonstrates the decrease of surfactant rejection along with the increase of surfactant/oil ratio. Particularly, for P and GO-P membranes, the decrease slope turns to be steeper as surfactant/oil ratio exceeding 0.15. This result also indicates that at excessively high surfactant/oil ratio, certain surfactant molecules exist in the form of self-aggregated micelle or even free molecule, which are much smaller in size compared with co-existed oil droplets and thus more difficult to be rejected. And only the membrane like GO-P-S with effective pore structure able to reject surfactant micelle can maintain a relatively stable removal of surfactant (~90%).

Generally, higher TMP will compress the foulants to be denser and thicker, strengthen their sticking on membrane surface, and thus result in the higher *R*_*fouling*_ for water molecules to pass through. Therefore, fouling becomes much severer as TMP elevated for all the three membranes (Fig. 6c-1). And owing to the multipotent antifouling capability of functionalized surface, GO-P-S membrane maintains the lowest fouling ratio under different operating pressures. Moreover, the remarkable increase in fouling ratio along with the elevation of TMP suggests that it’s not economically feasible to conduct the separation of nanoemulsions under TMP > 1 bar, especially for those underwater oleophilic membranes. For example, under TMP of 5 bar, the *J*_*W,nanoemulsion*_ of GO-P membrane (~78 L m^−2^ h^−1^) is even lower than that of P membrane (~84 L m^−2^ h^−1^), because this high TMP results in an extremely high fouling ratio (~97.1%) for GO-P membrane.

Figure 6c-2 displays that GO-P-S membrane is able to keep a stable oil rejection around 99.85% even when TMP is increased from 1 bar to 5 bar, while P and GO-P membranes suffer much severer losses in oil rejection. Taking account of the phenomenon that the aggravated membrane fouling along with the elevation of TMP can cause the apparent decrease in selective layer pore radius due to pore covering by foulants, it’s deduced pressure-squeezed oil intrusion is generated in the way that high pressure deforms nanometer-sized oil droplets and further squeeze them through membrane pores^51^. This also explains the corresponding decreases in surfactant rejection for P and GO-P membranes as shown in Fig. 6c-3, because the surfactant molecules bonded with oil is also brought through membrane along with pressure-squeezed oil intrusion. On the contrary, GO-P-S membrane maintains a relatively stable rejection of surfactant ~90.5% in spite of the elevation in TMP, which indicates its integrated surface properties contribute to resisting pressure-squeezed intrusions of oil and surfactant.

## Discussion

Membrane properties are listed in Table 1 to provide a comprehensive insight into the factors influencing fouling and concurrent rejections. And a clear trend of antifouling capability is revealed as GO-P-S membrane >P membrane >GO-P membrane. Firstly, GO-P membrane is poorer in antifouling property than P membrane. It’s the higher topography roughness that exacerbates fouling when membranes are similar in surface wettability. Secondly and more importantly, P membrane is outclassed by GO-P-S membrane in antifouling capability, though the two membranes have similar topography roughness. This indicates the synergistic effect between smooth topography and underwater oleophobicity is the key to acquire multipotent antifouling capabilities.

**Figure 7:**
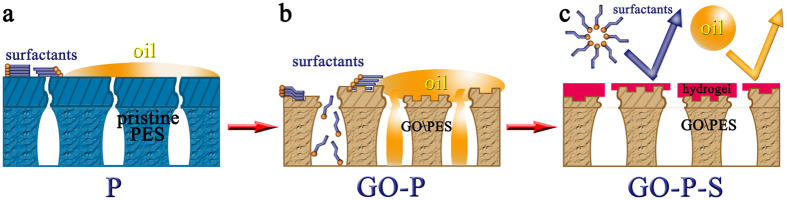
Different working mechanisms among P, GO-P and GO-P-S membranes during nanoemulsion separation process. (**a**) The hydrophobicity and underwater oleophilicity of P membrane leads to severe fouling induced by both surfactant and oil. (**b**) The nano-engineering by introducing GO sheet can increase pure water permeability (PWP) of phase inversion constructed membrane through enlarging selective layer pore radius. However, topography roughness of membrane is also increased with membrane surface remained oleophilic under water. As a result, GO-P membrane is poor in both antifouling capability and rejection of surfactant as well as oil. (**c**) Surface functionalization with hydrogel macromolecule can tune selective layer pore radius small enough to reject surfactant, reduce topography roughness and endow the resultant GO-P-S membrane with high oleophobicity under water. The synergistic effect of smooth topography and underwater oil-repellency leads to the multipotent antifouling capabilities that resist both oil and surfactant induced fouling. Moreover, GO-P-S membrane also achieves high rejections of both surfactant and oil.

Meanwhile, oil rejection follows the order of GO-P-S membrane >P membrane >GO-P membrane. Firstly, GO-P membrane is inferior in oil rejection to P membrane. This is because those big pores (~80 nm in diameter) of GO-P membrane are not effective to reject nanosized oil droplets especially under high TMP (Fig. 7a,b). Secondly and more importantly, GO-P-S membrane outperforms P membrane in oil rejection. This substantiates that surface wettability also has a significant impact on oil rejection besides pore size sieving effect. Our delicate surface functionalization contributes to preventing pressure-squeezed oil intrusion and thus leads to constantly higher oil rejection (Fig. 7c).

Furthermore, the concurrent rejection of surfactant follows the order of GO-P-S membrane >P membrane >GO-P membrane. This trend is consistent with the order of selective layer pore radius (*r*_*1,MWCO*_: GO-P-S membrane <P membrane <GO-P membrane). Therefore, it’s deduced that pore size exclusion plays an important role in surfactant rejection. And to effectively remove surfactant from nanoemulsion, pore radius smaller than or comparable to the Stokes radius of surfactant micelle is an essential requirement. Here, delicate surface functionalization is employed to fulfill this task through tuning selective layer pore radius down to <5 nm (*r*_*1,MWCO*_). Interestingly, GO-P-S membrane possesses higher rejection of surfactant and higher permeability compared with P membrane. This result substantiates that our nano-engineering technique is able to improve both membrane permeability and selectivity. Additional discussion including (1) membrane separation of nanoemulsion prepared from different kinds of oil, (2) membrane separation of nanoemulsion prepared from different surfactants, (3) nanoemulsion separation by membrane with different surface functionalizations, and (4) the comparison between this study and previous peer works on membrane separation of oil/water emulsion are elaborated in SI (Fig. S5–S7 and Table S3).

In conclusion, for the first time, the design rationale of nanoemulsion separating membrane is systematically elucidated in order to remove both surfactant and oil from nanoemulsion with low fouling and high flux. Guided by this design rationale, a hierarchical membrane with an ultrathin nanostructured selective layer on top of a microstructured support layer is successfully synthesized through the nano-engineering process: GO assisted phase inversion coupled with delicate surface functionalization. The nano-engineering by introducing GO sheet can enhance the permeability of phase inversion constructed membrane by five times through reducing selective layer thickness and increasing selective layer pore number density. The well-developed surface functionalization with an electroneutral hydrogel macromolecule can further fine-tune selective layer pore radius, smoothen membrane topography, and endow membrane with high oleophobicity underwater. As thus, the synergistic effect of smooth topography and oil-repellency leads to the multipotent antifouling capabilities that resist both surfactant and oil induced fouling. Compared with the membrane fabricated by conventional phase inversion process, nano-engineered GO-P-S membrane demonstrates four times higher water flux, constantly higher rejections of both oil droplets (~99.9%) and surfactant (as high as 93.5%) with only one third fouling ratio when separating oil-in-water nanoemulsion. To our best knowledge, this is the first nanoemulsion separating membrane that is able to address concurrent removal of surfactant and oil.

## Materials

### Chemicals

All chemicals were used as received. Polyethersulfone (PES, weight averaged molecular weight *M*_*w*_ ≈ 63 kDa, Solvay) and *N,N*-dimethylformamide (DMF, ≥ 99.8%, Sigma-Aldrich) were used as the polymer, and solvent, respectively, to prepare the polymer dope solution. GO nanosheet was synthesized via a modified Hummers’ method^52^,^53^. Polyvinyl alcohol (PVA, *M*_*w*_ ≈ 40.5 kDa, 99+% hydrolyzed, Sigma-Aldrich) was used as the electroneutral hydrogel macromolecule for surface functionalization^54^. Polyethylene glycol (PEG, Sigma-Aldrich) and polyethylene oxide (PEO, Sigma-Aldrich) were used as the neutrally charged solid solutes. Unless otherwise stated, a non-ionic surfactant (Triton X-100, Sigma-Aldrich) was purposely chosen in order to avoid the electrostatic interaction between membrane surface and surfactant complicating surfactant rejection, and a commercially available vegetable oil mixture (92% sunflower oil and 8% olive oil, see its detailed composition in SI) from DFI Brands was employed to prepare oil-in-water nanoemulsion.

### GO assisted phase inversion

Hierarchical flat sheet membrane was nano-engineered by GO assisted phase inversion technique. Here, weight fraction wt% refers to the proportion of entire dope solution. The nanocomposite dope solution was composed of 17.5 wt% PES, 0.5 wt% GO, and 82 wt% DMF. Firstly, as-synthesized graphite oxide was sonicated in DMF to obtain a homogenous GO solution. Secondly, PES was added into the GO solution under mechanical stirring. Thirdly, the mixture was heated at 50 °C while being stirred for 12 hours. Fourthly, a stainless steel knife (150 μm gate height) was driven by a film applicator (Elcometer, Belgium) at constant speed to cast the dope solution into a thin film. Finally, the cast film was immediately immersed in DI water (18 mΩ cm from Millipore system) to initialize GO assisted phase inversion at room temperature. The resultant nano-engineered membrane was stocked in 4 °C DI water before usage. Conventional phase inversion fabricated PES membrane was cast through the same procedure except that the GO weight fraction was zero.

### Delicate surface functionalization

Only the membrane fabricated through GO assisted phase inversion underwent this step. Firstly, hydrogel solution was prepared through dissolving PVA powder in DI water at 90 °C under mechanical stirring. The PVA concentration in hydrogel solution was ranged from 0 mg/L to 500 mg/L. Secondly, the membrane was sealed in a dead-end filtration module by rubber ring, wherein only membrane top surface is allowed to contact the hydrogel solution. The schematic diagram of filtration setup (diameter: 47 mm diameter, effective volume: 80 ml) is illustrated in Fig. S8. Thirdly, the deposition of hydrogel macromolecule was operated under 1 bar TMP for 20 min. Delicate surface functionalization was obtained through adjusting the concentration of hydrogel solution.

### Pure water permeability (PWP) and neutral solute selectivity

The PWP of membrane was tested under 1 bar TMP in dead-end filtration mode with DI water as feed solution. Molecular weight cutoff (MWCO, referring to the molecular weight of solute corresponding to 90% rejection by membrane) was determined according to solute transport method^55^, with PEG and PEO of different molecular weights used as solutes. Based upon the MWCO, membrane selective layer pore radius (*r*_*1,MWCO*_) is calculated according to the following equations, with detailed mathematical derivations provided in SI.

For MWCO tested from PEG,


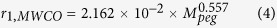


For MWCO tested from PEO,


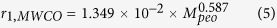


where *r*_*1,MWCO*_ is selective layer pore radius in the unit of nm that is calculated from MWCO, *M*_*peg*_ is MWCO of PEG in the unit of g/mol, *M*_*peo*_ is MWCO of PEO in the unit of g/mol.

### Preparation of oil-in-water nanoemulsions

Firstly, surfactant and oil were added into DI water sequentially under mechanical blending. Secondly, the mixture was sonicated in a bath sonicator (Branson) under relatively weak power (70 Watts) for 2 hours at 20 °C to obtain the initial emulsification. Thirdly, this nascent emulsion was further sonicated by a tip sonicator (Vibra Cell^TM^) under strong power (750 Watts) for 10 min to intensify the emulsification. Afterwards, as-prepared emulsion turned from opaque to semi-transparent, indicating that its oil droplet size was reduced from micrometer scale to nanometer scale (Fig. S9). Fresh nanoemulsion was immediately used as the feed solution for membrane separation.

### Membrane separation of nanoemulsion

Membrane separation of nanoemulsion was conducted in the dead-end filtration mode with the same filtration setup as described previously. Noteworthily, in order to minimize concentration polarization (CP), a mechanical agitator was stirred ~3 mm above membrane surface at 800 rpm during the separation process. Fouling ratio, the indicator of permeability loss due to fouling-induced resistance (see detailed mathematical analysis in SI), was calculated by equation 6.


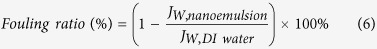


where *J*_*W,DI water*_ is pure water flux (L m^−2^ h^−1^) and *J*_*W,nanoemulsion*_ is the water flux with nanoemulsion as feed solution (L m^−2^ h^−1^). Here fouling ratio was recorded under 50% water recovery and reported as the average of parallel testing results of three pieces of membrane.

### Characterization

The morphology of as-synthesized GO nanosheet was characterized by transmission electron microscopy (TEM, JEOL 2010-H). Membrane structures were characterized by field emission scanning electron microscopy (FESEM, JEOL JSM 7600F). To acquire exposed cross-sections, membrane samples were immediately fractured after flash-frozen in liquid nitrogen. Membrane topography was probed by atomic force microscopy (AFM, Park XE-100) in non-contact mode. Membrane surface porosity is also calculated by gas adsorption-desorption method (Quadrasorb evo™, SI). Contact angles (AST products inc. VCA Optima) were probed by the sessile drop technique and reported as the average of 9 random measurements. 3 μl DI water in air or 15 μl 1,2-dicholoromethane under water were used as the probe liquids. All contact angle data were recorded at the initial moment when the probe liquid fully wet membrane surface. Dynamic laser light scattering (DLS, Mastersizer 2000) and optical microscopy (Olympus BX 60) were utilized to analyze the oil droplet size distribution of nanoemulsions. The turbidity data of nanoemulsions were recorded by HACH 2100N Turbidimeter. Total organic carbon (TOC, Shimadzu VCSH) and UV-Visible (Evolution 300 UV-Vis) were utilized to analyze oil and surfactant concentrations.

## Additional Information

**How to cite this article**: Qin, D. *et al*. A new nano-engineered hierarchical membrane for concurrent removal of surfactant and oil from oil-in-water nanoemulsion. *Sci. Rep*. **6**, 24365; doi: 10.1038/srep24365 (2016).

## Supplementary Material

Supplementary Information

## Figures and Tables

**Table 1:** Summary of membrane properties and fouling behaviour.

	P	GO-P	GO-P-S
Topography roughness (nm)	14.6 ± 2.6	32.8 ± 4.7	14.8 ± 3.1
MWCO (kDa)	65 ± 12	340 ± 41	15 ± 3
selective layer pore radius *r*_*1,MWCO*_ (nm)	10.4 ± 1.7	23.8 ± 2.9	4.6 ± 0.8
selective layer pore radius *r*_*1*_ (nm, FESEM)	~8	~17	~5
selective layer pore number density *N*_*1*_ (μm^−2^)	120 ± 20	410 ± 40	275 ± 30
selective layer thickness *L*_*1*_ (nm)	880 ± 90	490 ± 40	490 ± 55
membrane thickness *L*_*1*_ + *L*_*2*_ (μm)	68.0 ± 3	65.3 ± 2	65.4 ± 2
bottom surface pore radius (μm)	~0.3	~2.0	~2.0
water contact angle (°)	84.5 ± 2.3	64.5 ± 5.2	30.5 ± 3.3
underwater oil contact angle (°)	32.3 ± 3.5	67.4 ± 4.7	141.6 ± 3.5
pure water permeability (L m^−2^ h^−1^ bar^−1^)	102 ± 18	610 ± 62	162 ± 18
*J*_*W,nanoemulsion*_	GO-P-S > GO-P ≥ P		
Antifouling capability	GO-P-S > P > GO-P		
Oil rejection	GO-P-S > P > GO-P		
Surfactant rejection	GO-P-S > P > GO-P		
