# Supplementary material for: A new nano-engineered hierarchical membrane for concurrent removal of surfactant and oil from oil-in-water nanoemulsion
Source: Sci Rep. 2016 Apr 18;6:24365. doi: 10.1038/srep24365 (PMC4834549; doi:10.1038/srep24365)
Supplement: Supplementary Information [file srep24365-s1.pdf]

Supplementary Information (SI)

A new nano-engineered hierarchical membrane for concurrent removal of surfactant and oil from oil-in-water nanoemulsion

Detao Qin[1], Zhaoyang Liu[2,*], Hongwei Bai[3], Darren Delai Sun[4,*], and Xiaoxiao Song[4]

[1]Energy Research Institute @ NTU, Interdisciplinary Graduate School, Nanyang Technological University, 639798, Singapore

[2]Qatar Environment and Energy Research Institute (QEERI), Hamad bin Khalifa University (HBKU), Qatar Foundation, PO Box 5825, Doha, Qatar. E-mail: zhliu@qf.org.qa; Fax: +974 4454 0547; Tel: +974 4454 5621

[3]Energy Research Institute @ NTU, Nanyang Technological University, 639798, Singapore

[4]School of Civil and Environmental Engineering, Nanyang Technological University, 639798, Singapore. E-mail: ddsun@ntu.edu.sg; Fax: +65 6791 0676; Tel: +65 6790 6273

This file includes:

Page S2	Supplementary Introduction
Page S3~S20	Supplementary Results and Discussion.
Page S21~S23	Supplementary Experimental Details
Page S24~S25	Reference of SI

1. Supplementary Introduction

Table S1 | Typical hydraulic radius of various surfactants.

Surfactant type	surfactant	Stokes radius (concentration ≥ CMC)	Reference
Non-ionic	Triton X-100	1.8~11.9	1
		2.9~8.5	2
	Tween 80	3.7~3.9	3
	Eumulgin ES	~7.5	4
Ionic	SDS	4.5~5.0	5
	$CPClO_3$	3.0~7.5	6
	CPCl	~7.5	7

Note: CMC refers to critical micelle concentration, which is regarded as a charcteristic feature of surfactant. Surfactant is an amphiphilic compound that consists of both hydrophilic part and hydrophobic part. In polar solvents (*e.g.* water), the amphiphilic character of surfactant can lead to the self-aggregation phenomenon. The surfactant molecules arrange themsleves into an organized supramolecular assembly *i.e.* "micelle" with their hydrophibic parts aggregated into the core of micelle while their hydrophilic parts settled outwards in contact with water molecules to lower down the interfacial energy of micelle-water interface[8]. For each surfactant, there is a threshold of concentration below which surfactant molecules only exist in monomer form (*i.e.* free molecule) while above which micelle will form. And this concentration threshold is termed as critical micelle concentration (CMC). The Stokes radius of the micelle is related to both surfactant monomer radius and the number of monomers to form the micelle (*i.e.* the aggregation number, usually 10~100)[9]. Since monomer is much smaller than micelle in size, monomer is more difficult to be rejected by pore-flow membrane compared with micelle. This is also the reason that motivates this study to investigate the effect of surfactant concentration on the separation of surfactant-stabilized oil-in-water nanoemulsion by as-synthesized nanocomposite membrane (presented in the Results section of main text). SDS refers to sodium dodecyl sulfate, which is an anionic surfactant. $CPClO_3$ stands for cetylpyridinium chlorate while CPCl stands for cetylpyridinium chloride. Both $CPClO_3$ and CPCl are cationic surfactant.

2. Supplementary Results and Discussion

Elaborate mathematical analyses on the design of nanoemulsion separating membrane

(1) Tortuosity (τ)

Membrane tortuosity (τ) represents the ratio of the average distance for water molecules travelling through membrane to the thickness of membrane[10], as expressed in equation S1.

$$\tau = \frac{l}{L} \tag{S1}$$

where l is the average distance that water molecules take to travel through membrane, L is membrane thickness. In an ideal pore-flow membrane, the pores are in cylindrical shape that form parallel channels (perpendicular to membrane surface plane) connecting membrane top surface with bottom surface. For such ideal membrane, the value of τ is 1 (Figure S1-a). In reality, the value of τ is bigger than 1 (Figure S1-b). In other words, τ reflects the interconnectivity of pores in cross-sectional direction (perpendicular to membrane surface plane). The higher τ value, the lower pore interconnectivity is, and vice versa.

(2) Pore number density (N)

Pore number density (N) is defined as the number of pores per unit membrane area, which is in the unit of m^{-2} or μm^{-2}. The link between membrane surface porosity (ε) and pore number density (N) is expressed in equation S2.

$$\varepsilon = N\pi r_m^2 \tag{S2}$$

where ε is membrane surface porosity, r_m is membrane pore radius. This equation is based upon the assumption that the membrane pores are in circular shape.

(3) Hagen-Poiseuille equation

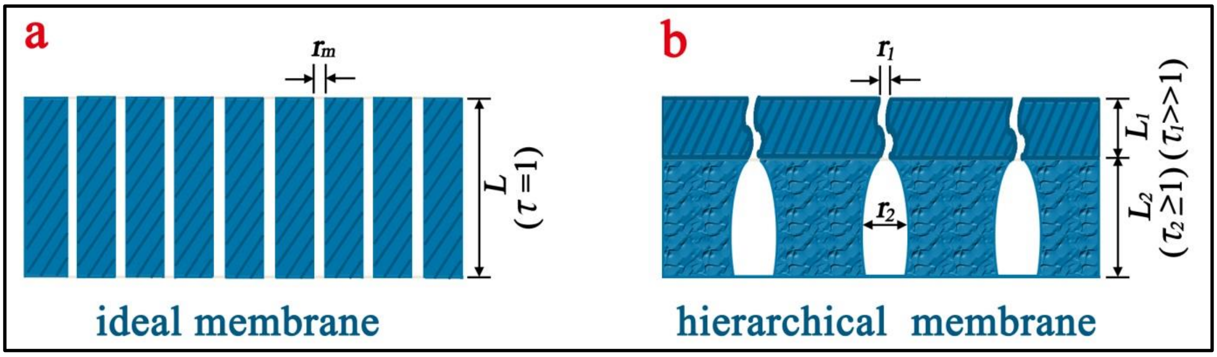

Figure S1 | Schematic diagram for the structure of (a) an ideal pore-flow membrane and (b) a hierarchical pore-flow membrane.

An ideal pore-flow membrane has isoporous structure as illustrated in Figure S1-a. According to Hagen-Poiseuille equation, the water flux of ideal pore-flow membrane can be expressed as follows[10].

$$J_{w,DI} = \frac{\varepsilon r_m^2}{8\eta} \times \frac{TMP}{\tau L} \quad \text{(S3)}$$

where $J_{W,DI}$ is pure water flux, η is the viscosity of DI water, and TMP is transmembrane pressure.

Equation S6 is obtained through substituting equation S2 into S3 and further dividing the resultant equation by TMP.

$$J_{w,DI} = \frac{N\pi r_m^2 \times r_m^2}{8\eta} \times \frac{TMP}{\tau L} \quad \text{(S4)}$$

$$\frac{J_{W,DI}}{TMP} = \frac{N\pi r_m^4}{8\eta} \times \frac{TMP}{\tau L} \times \frac{1}{TMP} \quad \text{(S5)}$$

$$PWP = \frac{\pi}{8\eta} \times \frac{N r_m^4}{\tau L} \quad \text{(S6)}$$

where PWP is pure water permeability of ideal pore-flow membrane.

The resistance induced by membrane itself for water molecules to pass through (R_m) can

be determined through using DI water as feed solution, as shown in equation S7.

$$R_m = \frac{TMP}{\eta \times J_{W,DI}} \quad \text{(S7)}$$

$J_{W,DI}$ can be expressed in equation S8 through the transformation of equation S7.

$$J_{W,DI} = \frac{TMP}{\eta \times R_m} \quad \text{(S8)}$$

Therefore, the expression of R_m as the function of membrane intrinsic properties can be obtained through substituting equation S8 into equation S4.

$$R_m = \frac{8\tau L}{\pi N r_m^4} \quad \text{(S9)}$$

For hierarchical pore-flow membrane (Figure S1-b), the resistance of selective layer R_1 and the resistance of support layer R_2 constitute the total resistance of membrane R_m (equation S10).

$$R_m = R_1 + R_2 \quad \text{(S10)}$$

Similarly, the expression of R_1 and R_2 as the function of membrane intrinsic properties can be obtained, as shown in equation S11 and S12, respectively.

$$R_1 = \frac{8\tau_1 L_1}{\pi N_1 r_1^4} \quad \text{(S11)}$$

where τ_1 is tortuosity of selective layer, L_1 is thickness of selective layer, N_1 is pore number density of selective layer, r_1 is average pore radius of selective layer.

$$R_2 = \frac{8\tau_2 L_2}{\pi N_2 r_2^4} \quad \text{(S12)}$$

where τ_2 is tortuosity of support layer, L_2 is thickness of support layer, N_2 is pore number density of support layer, r_2 is average pore radius of support layer.

Hence, R_m of hierarchical membrane can be expressed as equation S13.

$$R_m = \frac{8\tau_1 L_1}{\pi N_1 r_1^4} + \frac{8\tau_2 L_2}{\pi N_2 r_2^4} = \frac{8}{\pi}\left(\frac{\tau_1 L_1}{N_1 r_1^4} + \frac{\tau_2 L_2}{N_2 r_2^4}\right) \quad \text{(S13)}$$

Therefore, the PWP of hierarchical membrane can be obtained through substituting

equation S13 into equation S8 and further divided the resultant equation by TMP.

$$PWP = \frac{\pi}{8\eta}\frac{1}{(\frac{\tau_1 L_1}{N_1 r_1^4}+\frac{\tau_2 L_2}{N_2 r_2^4})} \tag{S14}$$

(4) Ferry equation

Membrane rejection of solute is dominated by the pore size of selective layer (r_1). In Ferry equation, the ratio of solute size (d_s) to selective layer pore size (d_1) is defined as λ.

$$\lambda = \frac{d_s}{d_1} = \frac{r_s}{r_1} \tag{S15}$$

where r_s is solute radius.

When $0 \leq \lambda \leq 1$ ($0 \leq r_s \leq r_1$), Ferry equation is defined as equation S16[11].

$$Rejection = [\lambda(2-\lambda)]^2 = [\frac{r_s}{r_1}\left(2-\frac{r_s}{r_1}\right)]^2 \tag{S16}$$

When $\lambda > 1$ ($0 \leq r_1 < r_s$), Ferry equation is defined as equation S17.

$$Rejection = 1 \tag{S17}$$

The plot of rejection as the function of λ is shown in Figure S2 according to Ferry equation. It's clear that rejection is increased monotonously along with the increase of λ within the range of $0 \leq \lambda \leq 1$ ($0 \leq r_s \leq r_1$). Therefore, given at a certain r_s value, the increase of r_1 leads to the decrease of λ and thus the decrease of rejection. This quantifies the negative correlation between r_1 and membrane rejection.

Noteworthily, Ferry equation is based upon the assumptions that (1) membrane rejection relies on size exclusion principle, (2) there is no interaction (*e.g.* electrostatic repulsion) between membrane surface and solute, and (3) the shape of solute is fixed, particularly independent on solute concentration. However, for the virtual nanoemulsion separation process, the applicability of this equation is limited. This is because unlike solid particles, oil

droplets are soft and can be deformed under pressure, resulting in the squeezing of oil through membrane pores that are smaller than oil droplet in size[12]. Moreover, both oil and surfactant can cause severe membrane fouling in the form of pore-plugging or cake-forming. Therefore, the virtual rejection can be smaller than the value predicted from Ferry equation. Here, Ferry equation only provides the negative correlation between r_1 and membrane rejection in a simplified way, which indicates that larger r_1 generally leads to smaller rejection.

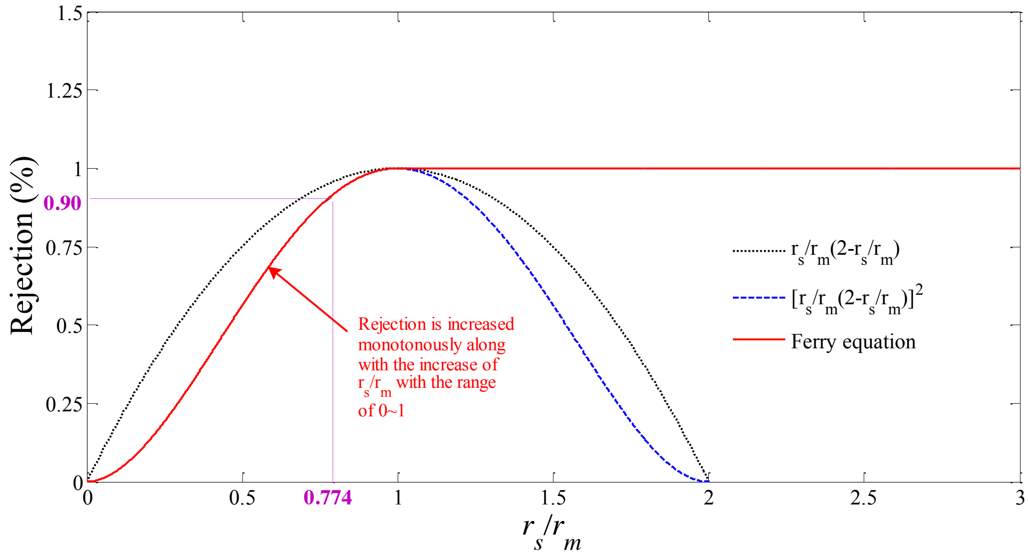

Figure S2 | The graph of Ferry equation.

(5) Theoretically maximum water flux of hierarchical membrane during nanoemulsion separation process

Membrane antifouling property can be evaluated via the resistances-in-series model[13], which is represented mathematically by equation S18 when concentration polarization (CP) is negligible.

$$R_t = R_m + R_{fouling} \tag{S18}$$

where R_t is the total resistance, R_m is the resistance induced by membrane itself, and $R_{fouling}$ is fouling induced resistance respectively.

During the separation of oil-in-water nanoemulsion, the breakthrough pressure is the minimum pressure at which the oil droplet will be pushed through the pore of membrane despite otherwise unfavorable wetting property and geometrical constraints. And the breakthrough pressure follows Young-Laplace equation given that selective layer pore radius is smaller than oil droplet radius[14].

$$P_{breakthrough} = \frac{2\gamma_{ow}cos\theta}{d_1} = \frac{\gamma_{ow}cos\theta}{r_1} \quad \text{(S19)}$$

where γ_{ow} is oil-water interfacial tension, θ is oil contact angle of surface in the presence of water, d_1 is pore diameter of selective layer.

And the water flux with nanoemulsion as feed solution ($J_{W,\ nanoemulsion}$) can be expressed by equation S20 according to resistances-in-series model.

$$J_{W,nanoemulsion} = \frac{TMP}{\eta \times R_t} = \frac{TMP}{\eta \times (R_m + R_{fouling})} \quad \text{(S20)}$$

Therefore, theoretically maximum $J_{W,\ nanoemulsion}$ of hierarchical membrane can be obtained by setting TMP equal to breakthrough pressure. Accordingly, equation 4 in main text is obtained through substituting equation S13 and S19 into S20.

$$J_{w,nanoemulsion} = \frac{\gamma_{ow}cos\theta}{\eta r_1(\frac{8}{\pi}(\frac{\tau_1 L_1}{N_1 r_1^4} + \frac{\tau_2 L_2}{N_2 r_2^4}) + R_{fouling})} \quad \text{(S21)}$$

Noteworthily, $R_{fouling}$ is determined by the interactions between foulants and membrane. Particularly, membrane pore structure can influence membrane-foulant interactions. For example, when selective layer pore radius r_1 is larger than foulant radius, internal fouling can be triggered in the way that foulants enter membrane pores and plug these pores. On the contrary, when r_1 is smaller than foulant radius, membrane fouling takes place mainly in the

way of external fouling that foulants only deposit on membrane surface. External fouling is more reversible compared with internal fouling, because external fouling can be mitigated through washing membrane surface while internal fouling cannot be mitigated by this method. In short, $R_{fouling}$ can be regarded as a function of r_1.

(6) The positive correlation between fouling induced resistance ($R_{fouling}$) and fouling ratio (FR).

In this study, fouling ratio (FR) is utilized as the indicator of fouling induced resistance ($R_{fouling}$). The merits by doing so include (1) to make the characterization of membrane fouling easy and simple, and (2) to provide the direct link between flux decline and $R_{fouling}$. Noteworthily, the effect of concentration polarization is minimized through increasing flow turbulence on membrane surface, and hence R_t consists of only two parts R_m and $R_{fouling}$, as aforementioned in equation S18. The expression of R_t can be obtained through the transformation of equation S20.

$$R_t = \frac{TMP}{\eta \times J_{W,nanoemulsion}} \quad \text{(S22)}$$

The expression of $J_{W,\ nanoemulsion}$ as the function of FR can be obtained through conducting the transformation of equation 6 of main text.

$$J_{w,nanoemulsion} = (1 - FR) \times J_{W,DI} \quad \text{(S23)}$$

Equation S24 is obtained through substituting equation S18 and S23 into equation S22.

$$R_m + R_{fouling} = \frac{TMP}{\eta \times (1 - FR) \times J_{W,DI}} \quad \text{(S24)}$$

Equation S25 is obtained through substituting equation S7 into equation S24.

$$R_m + R_{fouling} = \frac{R_m}{1 - FR} \quad \text{(S25)}$$

Therefore, the correlation between FR and R_m can be obtained through conducting the following transformations of equation S25.

$$1 - FR = \frac{R_m}{R_m + R_{fouling}} \quad \text{(S26)}$$

$$FR = \frac{R_{fouling}}{R_m + R_{fouling}} \quad \text{(S27)}$$

$$FR = \frac{1}{\frac{R_m}{R_{fouling}} + 1} \quad \text{(S28)}$$

For a given type of membrane, R_m is a constant value. Therefore, FR can be regarded as a function of only one variable '$R_{fouling}$'. More importantly, equation S28 clearly demonstrates the positive correlation between $R_{fouling}$ and FR. In short, FR can be used as the direct indicator of fouling induced resistance (R_m).

(7) The calculation of selective layer pore radius $r_{1,MWCO}$ based upon membrane rejection performance.

In this study, polyethylene glycol (PEG) and polyethylene oxide (PEO) are used for the evaluation of membrane selectivity. Stokes radius (hydraulic radius) of PEG and PEO can be calculated from their molecular weight according to the following equations[15].

For PEG,

$$r_{s,peg} = 1.673 \times 10^{-2} \times M_{peg}^{0.557} \quad \text{(S29)}$$

where $r_{s,peg}$ is the Stokes radius of PEG in the unit of nm; M_{peg} is the molecular weight of PEG, in the unit of g/mol.

For PEO,

$$r_{s,peo} = 1.044 \times 10^{-2} \times M_{peo}^{0.587} \quad \text{(S30)}$$

where $r_{s,peo}$ is Stokes radius of PEO in the unit of nm; M_{peg} is the molecular weight of PEO in

the unit of g/mol.

Molecular weight cutoff (MWCO) was determined according to solute transport method[16], with different molecular weights of PEG and PEO used as feed solutions. The value of MWCO refers to the molecular weight of solute corresponding to membrane rejection of 90%. Therefore, Stokes radius of PEG and PEO corresponding to MWCO can be obtained based upon equation S29 ~ S30 by setting M_{peg} or M_{peg} equal to MWCO.

Considering PEG and PEO are neutrally charged solutes, Ferry equation is applicable to model the correlation between selective layer pore radius r_1 and hydraulic radius of solute r_s in a simplified way. By setting membrane rejection equal to 90%, the ratio of r_s/r_1 (λ) can be solved out from equation S16, with the result shown in equation S31.

$$\lambda = \frac{r_s}{r_1} = 0.774 \ \ when\, rejection = 90\% \qquad \text{(S31)}$$

Therefore, membrane selective layer pore radius can be calculated based upon MWCO through substituting equation S29 or S30 into equation S31.

For MWCO tested from PEG,

$$r_{1,MWCO} = \frac{r_{s,peg}}{0.774} = 2.162 \times 10^{-2} \times M_{peg}^{0.557} \qquad \text{(S32)}$$

For MWCO tested from PEO,

$$r_{1,MWCO} = \frac{r_{s,peo}}{0.774} = 1.349 \times 10^{-2} \times M_{peo}^{0.587} \qquad \text{(S33)}$$

where $r_{1,MWCO}$ is selective layer pore radius (membrane top surface pore radius) calculated from MWCO in the unit of nm.

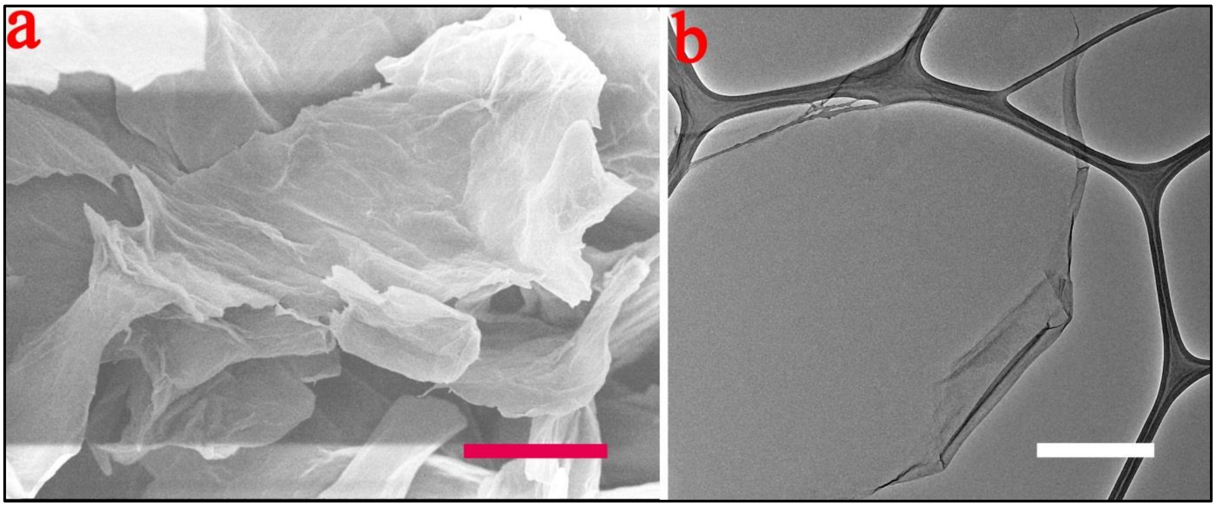

Figure S3 | The characterizations of as-synthesized GO sheet. (a) FESEM image of as-synthesized graphite oxide (scale bar, 5 μm). Plenty of wrinkles are formed on the microplates of as-synthesized graphite oxide, indicating the crystal structure of graphite is disturbed by the intercalation (of concentrated H_2SO_4[17]) and oxidation (with $KMnO_4$ and H_2O_2 in the presence of $NaNO_3$) processes during the synthetic steps. (b) TEM image of a single layer graphene oxide sheet (scale bar, 500 nm). The central part of graphene oxide monolayer appears almost transparent owing to its nanometer scale thickness while its edges form curls and foldings due to uneven mechanical support from the sample holder (copper grid).

Table S2 | Membrane selective layer (top surface) pore number density measured by gas adsorption-desorption method.

	P membrane	GO-P membrane	GO-P-S membrane
Typical Pore radius distribution curve	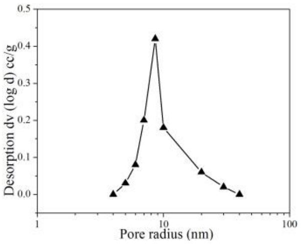	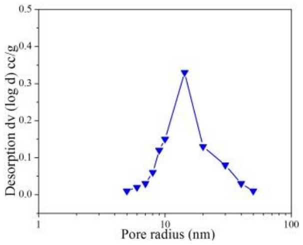	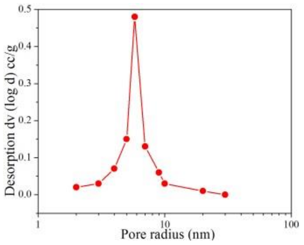
Average selective layer pore radius $r_{1,gas}$ (nm)	8.6	14.3	5.8
Surface porosity ε_1 (cm^2/g)	1.05 ± 0.17	5.98 ± 0.79	0.68 ± 0.10
Surface porosity ε_1 (10^{-2} cm^2/cm^2, %)	1.31 ± 0.21	7.26 ± 0.96	0.86 ± 0.12
Selective layer pore number density $N_{1,gas}$ (μm^{-2})	56 ± 9	113 ± 15	81 ± 11

Note: the experimental procedure contains multiple steps in sequence as follows: (1) freeze-dry membrane samples, (2) cut the sample into a square shape with the lateral size of 3 cm × 3 cm (area is 9 cm^2), (3) measure the weight of this square shape membrane sample (*e.g.* ~0.1 g), (4) further cut this membrane sample into smaller pieces that can be loaded into testing tube, (5) conduct gas adsorption-desorption experiment using nitrogen gas (Quadrasorb evo^{TM}), (6) obtain the porosity result in the unit of cm^2/g sample (BET calculation method), (7) convert the result into surface porosity in the unit of cm^2/cm^2 sample, (8) calculate surface pore number density based upon equation S2: $N_1 = \varepsilon_1 / (\pi \times r_1^2)$. This method takes account of that only the top surface of as-synthesized membranes is in mesoporous structure (2~50 nm, IUPAC). Noteworthily, the selective layer pore number density calculated indirectly from gas adsorption-desorption method ($N_{1,gas}$) is considerably smaller than that is estimated directly from FESEM image. This is possibly because FESEM image cannot differentiate well effective pores (surface pore that is connected with sub-surface pores) from dead pores (false surface pore that is not connected with sub-surface pores). Moreover, $N_{1,gas}$ follows the order of GO-P membrane > GO-P-S membrane > P membrane. This trend is consistent with FESEM observations as presented in main text.

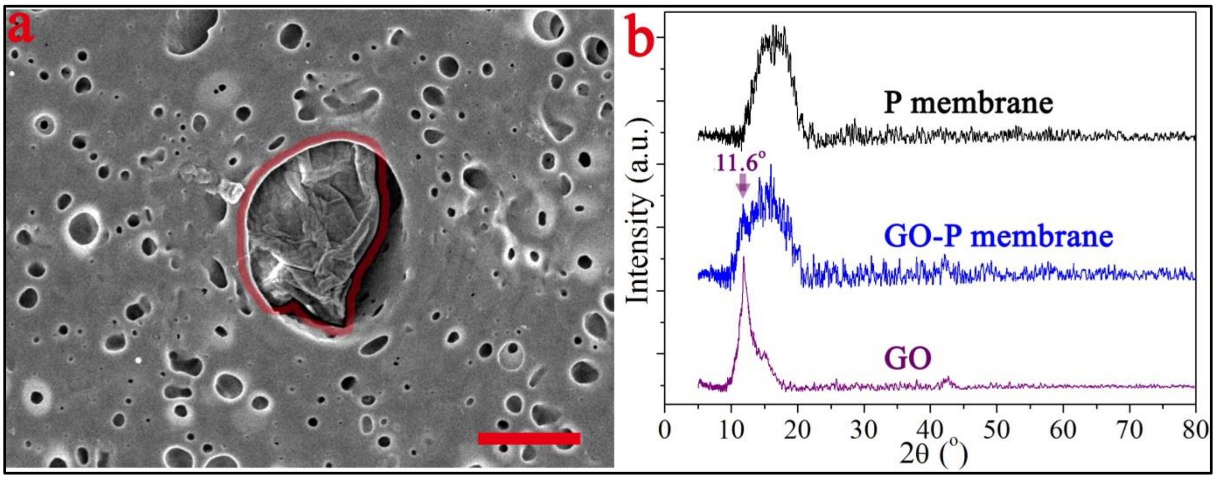

Figure S4 | The existence of GO nanosheet in as-synthesized nanocomposite membranes. (**a**) Enlarged FESEM image on GO-P-S membrane (bottom surface, scale bar, 2 μm). The red circle highlights the embedded GO nanosheets. (**b**) XRD pattern of P membrane, GO-P membrane and GO, respectively. The purple arrow indicates that there exists a characteristic peak at 11.6° on the XRD pattern of GO-P membrane, which originates from the XRD pattern of GO nanosheets embedded into the nanocomposite membrane[18] and is totally absent from the XRD pattern of the P membrane alone. Hence these XRD characterization results also confirm the presence of GO in the matrix of as-synthesized nanocomposite membranes.

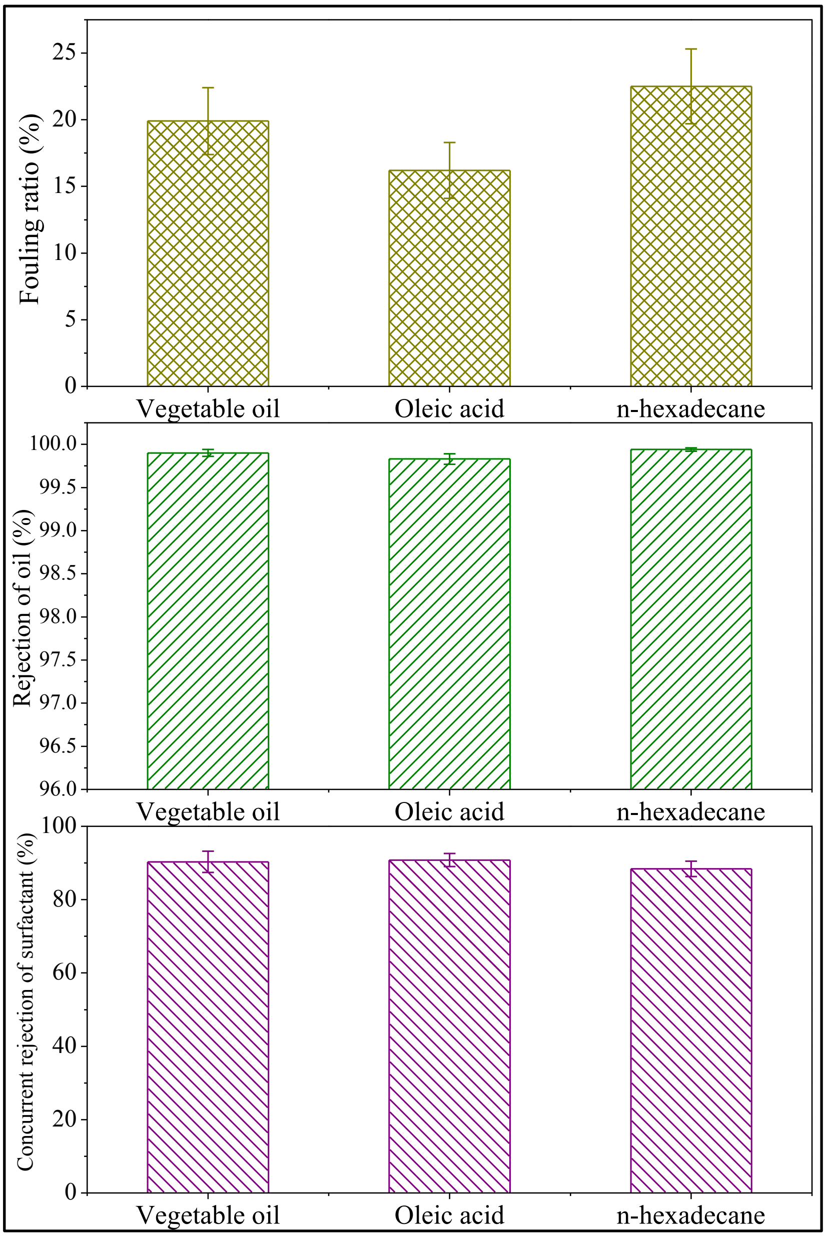

Figure S5 | The separation of nanoemulsion prepared from different kinds of oil by GO-P-S membrane. The oil concentration is 1200 mg/L and the surfactant/oil ratio is 0.15. The detailed composition of vegetable oil is presented in Table S4. Oleic acid (≥99% purity) and *n*-hexadecane (≥99% purity) are purchased from Sigma-Aldrich. This figure shows that the fouling ratio of GO-P-S membrane is 16.2% ~ 22.5%; the oil rejection by GO-P-S membrane is >99.80%; and the concurrent rejection of surfactant by GO-P-S membrane is >90.0%. These results demonstrate that GO-P-S membrane can achieve good performance when investigated with oil-in-water nanoemulsion prepared from different kinds of oil.

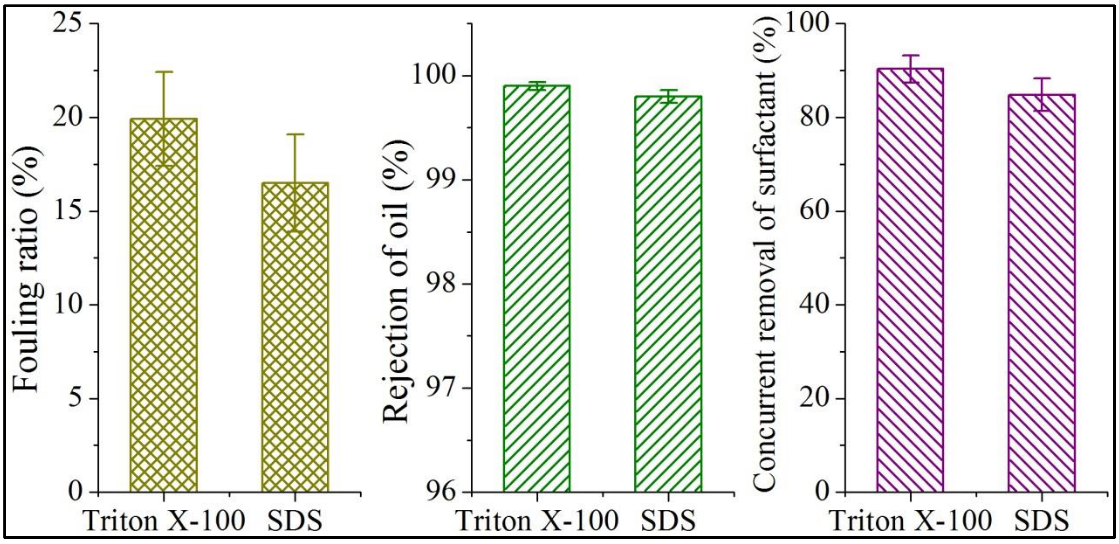

Figure S6 | The separation of nanoemulsion prepared from different surfactants by GO-P-S membrane. The oil concentration is 1200 mg/L and the surfactant/oil ratio is 0.15. SDS stands for ionic surfactant sodium dodecyl sulfate. The fouling ratio of GO-P-S membrane is lower under the emulsion prepared with ionic surfactant SDS (16.5% ± 2.6%) than that with non-ionic surfactant Triton X-100 (19.8% ± 2.5%). Remarkably high rejections of oil by GO-P-S membrane can be achieved for the emulsion prepared with each surfactant separately (both >99.8%). The concurrent rejection of surfactant by GO-P-S membrane is higher under the emulsion prepared with Triton X-100 (90.3% ± 2.9%) than that with SDS (85.1% ± 3.4%). Moreover, the total organic carbon (TOC) concentration of the permeate for the emulsion prepared with each surfactant separately is lower than 30 ppm, which meets the discharge standards on oil & gas produced wastewater by U.S.A. Environmental Protection Agency (40 CFR Part 435)[19].

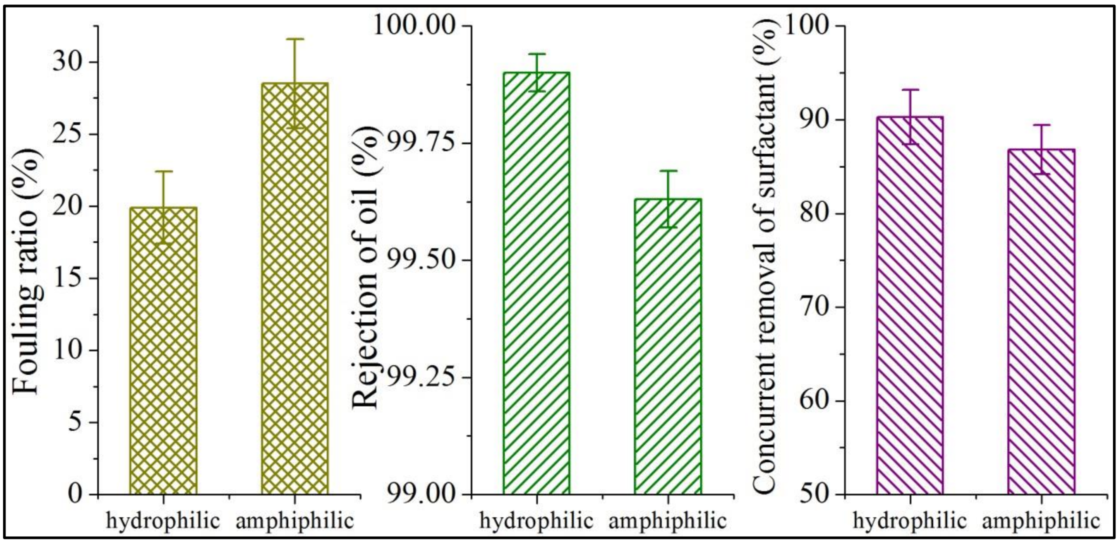

Figure S7 | The comparison between hydrophilic surface modification (GO-P-S membrane) and amphiphilic surface modification (GO-P-Sa membrane). The amphiphilic polymer used for surface modification is PBMA-*g*-PEGMA, which is synthesized through grafting hydrophilic polymer poly(ethylene glycol) methacrylate (PEGMA, Sigma-Aldrich) on hydrophobic backbone poly(butyl methacrylate) (PMBA, Sigma-Aldrich) through free radical polymerization. The experimental details about grafting polymerization follow the method of published article by Liu Y *et al*.[20]. The amphiphilic modification of GO-P membrane employs the same procedure with hydrophilic modification. And as-synthesized membrane with amphiphilic surface modification is termed as GO-P-Sa membrane, wherein "a" refers to amphiphilic modification. The water contact angle of GO-P-Sa membrane is 61° ± 3.3° and the underwater oil contact angle of GO-P-Sa membrane is 55° ± 3.4°. Through controlling the concentration of PBMA-*g*-PEGMA solution, the pure water permeability of GO-P-Sa membrane (156 ± 10 L m^{-2} h^{-1} bar^{-1}) is purposely adjusted to be nearly the same with that of GO-P-S membrane (162 ± 18 L m^{-2} h^{-1} bar^{-1}). Figure S6 indicates that GO-P-S membrane possesses better antifouling capability, higher oil rejection and higher concurrent surfactant rejection than GO-P-Sa membrane during the separation of oil-in-water nanoemulsion (the oil concentration is 1200 mg/L and the surfactant/oil ratio is 0.15). The relatively poor fouling-mitigation of GO-P-Sa membrane is mainly attributed to its (underwater) oleophilicity. This result also indicates that GO-P-S membrane is the best membrane among all the membranes synthesized to separate oil-in-water nanoemulsion.

Table S3 | The comparison on membrane separation of oil/water emulsion between this study and previous works.

Oil/water emulsions		Membrane surface wettability	Membrane thickness (μm)	Pore diameter (nm)	Pure solvent permeability ($Lm^{-2}h^{-1}bar^{-1}$)	Fouling ratio (%)	Droplet rejection (%)	Simultaneous rejection of surfactant (%)	Ref.
Type	Size								
water-in-oil	microemulsion	hydrophobic	50	~100*	~10^4	20[a]	99.95	N.S.	[21]
oil-in-water	microemulsion	**amphi**philic	235*	77	200	31.1	N.S.	N.S.	[20]
oil-in-water	microemulsion	hydrophilic	188	18.6	191	46.2	98.8	N.S.	[22]
oil-in-water	microemulsion	hydrophilic	180*	136	20.5	40	98	N.S.	[23]
oil-in-water	microemulsion	hydrophilic	176*	200~750	7500	46.7	99	N.S.	[24]
water-in-oil	**nano**emulsion	hydrophobic	169*	30~80[b]	375	N.S.	99*	N.S.	[25]
oil-in-water	**nano**emulsion	**amphi**philic	230*	N.S.	157	78.4	98.9	N.S.	[26]
oil-in-water	**nano**emulsion	hydrophilic	N.S.	6.84	24.3	30	96.6	N.S.	[27]
oil-in-water	**nano**emulsion	hydrophilic	0.077	~25*	35,000~61,000	85*	99.99	N.S	[28]
oil-in-water	**nano**emulsion	hydrophilic	65.4	9.2	162	19.5	99.9	89.3~93.5	**this study**

Note: N.S. means the data has not been studied by the research article. "*" means no direct data is provided by the corresponding article and hence the number listed in the table is estimated from the other relevant information (*e.g*. SEM image) of that article. "**a**" means the data is calculated after 20 cycles' operation, with ethanol used in-between each cycle to clean the membrane to recover solvent flux. "**b**" means the pore diameter value is before surface coating.

Additional discussion on Table S3.

Table S3 compares membrane separation of oil/water emulsion between this study and previous works. Five points need to be emphasized as follows.

Firstly, both the study from Zhang W *et al.*[21] and the study from Solomon B *et al.*[25] synthesized the membrane with hydrophobic surface to separate water-in-oil emulsion. Oil is allowed to permeate through this kind of membrane while water droplets are retained owing to its surface hydrophobicity. However, this kind of membrane has several intrinsic problems. For example, the flux of this kind of membrane is limited at high solvent (oil) viscosity, according to Hagen-Poiseuille equation[10].

Secondly, the solvent flux during the separation of oil/water emulsion is noticeably high for the study from Zhang W *et al.*[21] (~10^4 L m^{-2} h^{-1} bar^{-1}) and the study from Chen P *et al.*[24] (7,500 L m^{-2} h^{-1} bar^{-1}). This is mainly because the surface pore diameter of their membranes is 1~2 orders of magnitude larger than that of GO-P-S membrane synthesized in this study. However, the rejection of solid solute by membrane has a negative correlation with membrane pore size, according to Ferry equation[11]. And these two membranes are designed primarily for the separation of microsized oil/water emulsions but not for the rejection of nanosized pollutants (*e.g*. surfactant micelles).

Thirdly, Hu L *et al*. used single walled carbon nanotube derived nanomaterial to fabricate a nanoporous membrane to separate oil-in-water nanoemulsion[28]. This membrane has ultrahigh pure water permeability of 35,000 ~ 61,000 L m^{-2} h^{-1} bar^{-1}, which is mainly because the thickness of this membrane is as thin as only 77 ± 25 nm. However, it's worthy to note that the ultrathin thickness inevitably compromises the mechanical strength of this kind of membranes, which adversely affect their practical applicability. Moreover, a considerably high fouling ratio is observed for this membrane, though a high rejection of nanosized oil droplets can be obtained.

Fourthly, our GO-P-S membrane demonstrates the remarkable advantage in terms of antifouling capability over most previous works listed in Table S2, as evidenced by that the fouling ratio of GO-P-S membrane is the lowest. Particularly, Liu Y *et al*.[20] and Rajasekhar T *et al*.[26] conducted respective research to use the polymeric membrane with amphiphilic surface modification to separate surfactant-stabilized oil-in-water emulsion. And the fouling results observed in their amphiphilic membranes are considerably heavier than that of our GO-P-S membrane. This is also in agreement with our result that the antifouling capability of hydrophilic (oleophobic) surface functionalized nanocomposite membrane (GO-P-S membrane) is better than that of amphiphilic surface functionalized nanocomposite membrane (GO-P-Sa membrane).

Fifthly and most importantly, this study for the first time clearly demonstrates that concurrent high rejection of surfactant can be achieved besides high rejection of nanosized oil droplet, using our deliberately designed nanocomposite membrane (GO-P-S membrane).

3. Supplementary Experimental Details

Table S4 | Detailed composition of the vegetable oil used in this study.

	Average quantity per 1 L
Energy	34,160 kJ<br>8,160 kcal
Protein	0.0 g
Fat, total	900 g
——saturated fat	107 g
——trans fat	6 g
Cholesterol	0 mg
Carbohydrate	7 g
Sugars, total	0.0 g
Dietary fiber	0.0 g
Sodium	0 mg
Calcium	1 mg

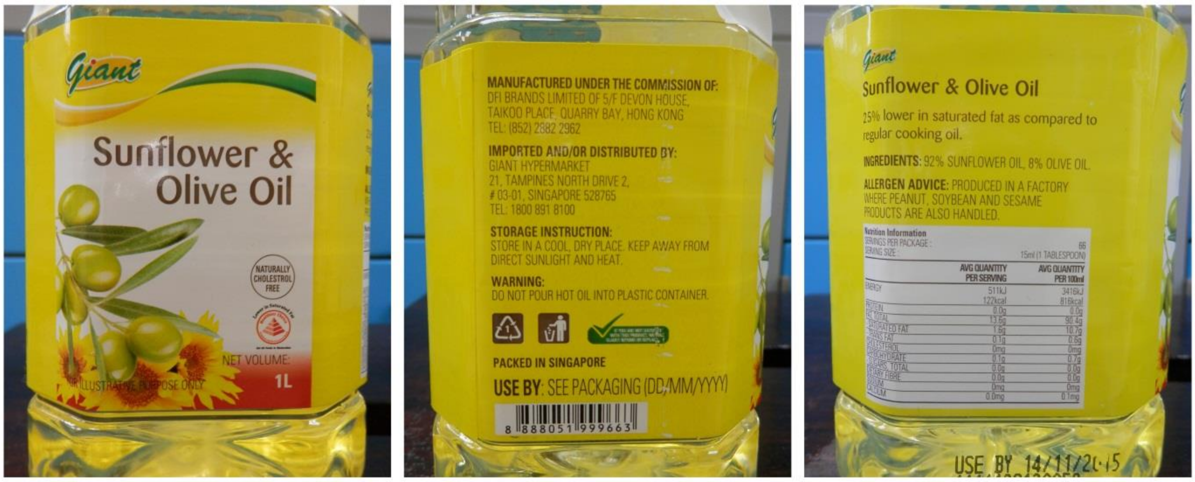

Note: this table is quoted from the product label, which is shown in the inserted photos. This vegetable oil is purchased from a local supermarket named "*Giant*". The brand name of this vegetable oil is "*Sunflower & Olive Oil*" (92% sunflower oil and 8% olive oil). And the producer of this oil is *DFI Brands Limited Hong Kong*.

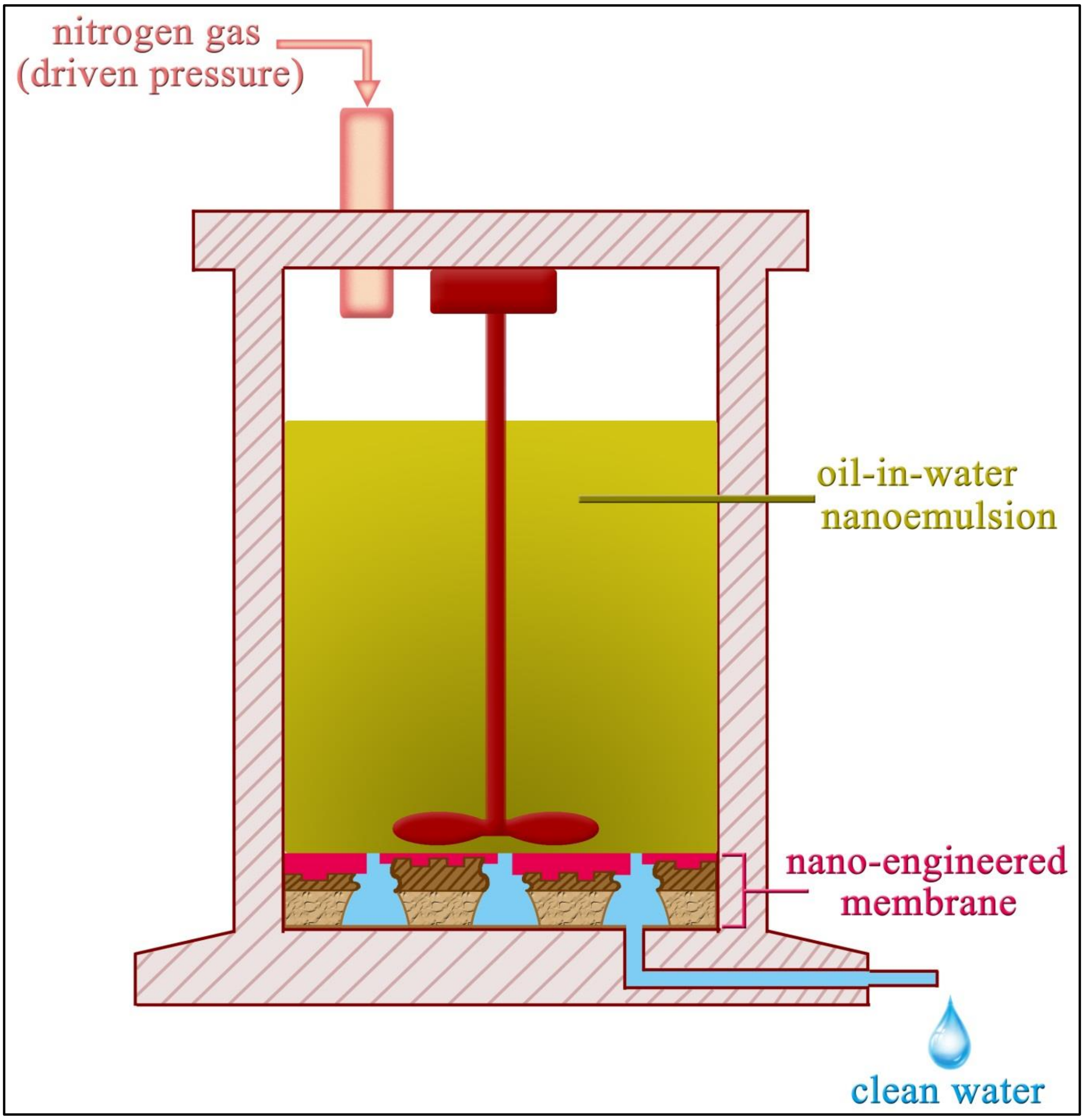

Figure S8 | Schematic diagram of the filtration setup. In order to mitigate concentration polarization (CP), a mechanical agitator was stirred ~3 mm above membrane surface at 800 rpm during the separation of oil-in-water nanoemulsion.

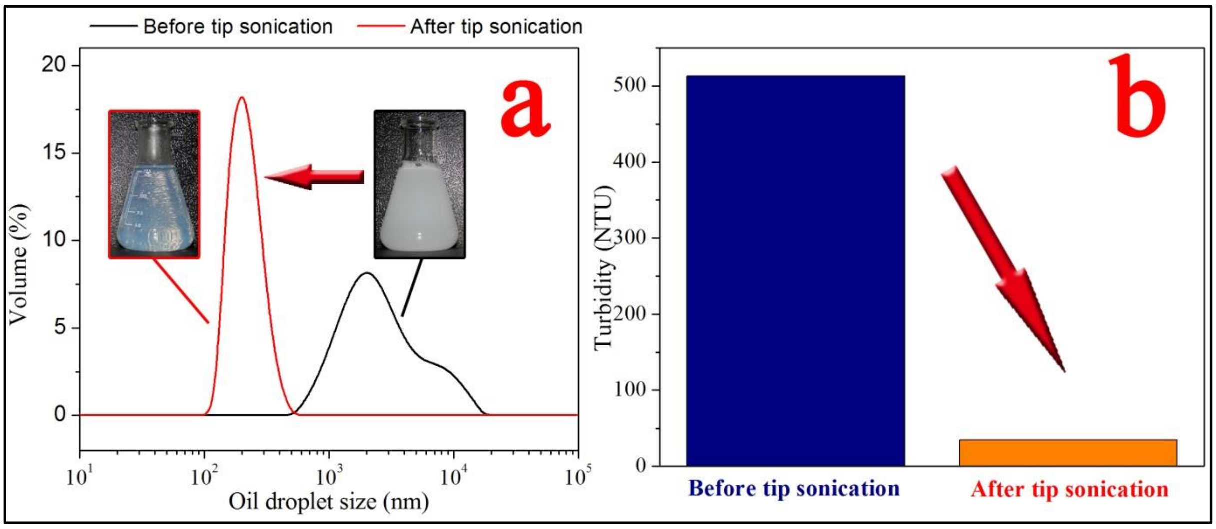

Figure S9 | The effect of tip sonication on oil droplet size distribution. (a) Dynamic laser light scattering (DLS) data of the same oil-in-water emulsion before and after tip sonication, respectively. The tip sonication at strong power (750 W) could reduce oil droplet size from micrometer scale to nanometer scale. The inset pictures are the optical photo of the same emulsion before and after tip sonication, respectively. (b) Turbidity data of the same oil-in-water emulsion before and after tip sonication, respectively. The oil concentration is 1200 mg/L with 0.15 surfactant/oil ratio.

References of SI

1 Paradies, H. H. Shape and size of a non-ionic surfactant micelle Triton X-100 in aqueous solution. *J. Phys. Chem.* **84**, 599-607 (1980).

2 Streletzky, K. & Phillies, G. D. J. Temperature dependence of Triton X-100 micelle size and hydration. *Langmuir* **11**, 42-47 (1995).

3 Singh, R. Investigation of ultrafiltration rejection of surfactant micelles by dynamic light scattering. *Sep. Sci. Technol.* **31**, 1351-1356 (1996).

4 Fischer, I. & Franzreb, M. Removal of the nonionic surfactant Eumulgin ES from protein solutions by means of adsorption and ultrafiltration. *Sep. Purif. Technol.* **118**, 217-225 (2013).

5 Mazer, N. A., Benedek, G. B. & Carey, M. C. Investigation of micellar phase of sodium dodecyl sulfate in aqueous sodium chloride solutions using quasi elastic light scattering spectrocopy. *J. Phys. Chem.* **80**, 1075-1085 (1976).

6 Myska, J., Stepanek, P. & Zakin, J. L. Micellar size of drag reducing cationic surfactants. *Colloid Polym. Sci.* **275**, 254-262 (1997).

7 Berret, J. F., Appell, J. & Porte, G. linear rheology of entangled wormlike micelles. *Langmuir* **9**, 2851-2854 (1993).

8 Dominguez, A., Fernandez, A., Gonzalez, N., Iglesias, E. & Montenegro, L. Determination of critical micelle concentration of some surfactants by three techniques. *J. Chem. Educ.* **74**, 1227-1231 (1997).

9 Nesmerak, K. & Nemcova, I. Determination of critical micelle concentration by electrochemical means. *Anal. Lett.* **39**, 1023-1040 (2006).

10 Robinson, J. P., Tarleton, E. S., Millington, C. R. & Nijmeijer, A. Solvent flux through dense polymeric nanofiltration membranes. *J. Membr. Sci.* **230**, 29-37 (2004).

11 Ferry, J. D. Ultrafilter membranes and ultrafiltration. *Chem. Rev.* **18**, 373-455 (1936).

12 Cobos, S., Carvalho, M. S. & Alvarado, V. Flow of oil-water emulsions through a constricted capillary. *Int. J. Multiph. Flow* **35**, 507-515 (2009).

13 Tansel, B., Bao, W. Y. & Tansel, I. N. Characterization of fouling kinetics in ultrafiltration systems by resistances in series model. *Desalination* **129**, 7-14 (2000).

14 Xue, Z. X. *et al*. A novel superhydrophilic and underwater superoleophobic hydrogel-coated mesh for oil/water separation. *Adv. Mater.* **23**, 4270-4273 (2011).

15 Singh, S., Khulbe, K. C., Matsuura, T. & Ramamurthy, P. Membrane characterization by solute transport and atomic force microscopy. *J. Membr. Sci.* **142**, 111-127 (1998).

16 Zhang, X. W., Zhang, T., Ng, J. & Sun, D. D. High-performance multifunctional TiO2 nanowire ultrafiltration membrane with a hierarchical layer structure for water treatment. *Adv. Funct. Mater.* **19**, 3731-3736 (2009).

17 Hong, Y. Z., Wang, Z. Y. & Jin, X. B. Sulfuric Acid Intercalated Graphite Oxide for Graphene Preparation. *Sci Rep* **3** (2013).

18 Marcano, D. C. *et al*. Improved synthesis of graphene oxide. *Acs Nano* **4**, 4806-4814 (2010).

19 U. S. Environmental Protection Agency., Final effluent limitations guidelines and standards for the coastal subcategory of the oil and gas extraction point source category. (1996). Available at: http://www.gpo.gov/fdsys/pkg/FR-1996-12-16/pdf/96-28659.pdf. Date of access: 21/11/2015.

20 Liu, Y., Su, Y. L., Li, Y. F., Zhao, X. T. & Jiang, Z. Y. Improved antifouling property of PVDF membranes by incorporating an amphiphilic block-like copolymer for oil/water emulsion separation. *RSC Adv.* **5**, 21349-21359 (2015).

21 Zhang, W. B. *et al*. Superhydrophobic and superoleophilic PVDF membranes for effective separation of water-in-oil emulsions with high flux. *Adv. Mater.* **25**, 2071-2076 (2013).

22 Luo, L. *et al*. Oil/water separation via ultrafiltration by novel triangle-shape tri-bore hollow fiber membranes from sulfonated polyphenylenesulfone. *J. Membr. Sci*. **476**, 162-170 (2015).

23 Zhu, X. Y., Tu, W. T., Wee, K. H. & Bai, R. B. Effective and low fouling oil/water separation by a novel hollow fiber membrane with both hydrophilic and oleophobic surface properties. *J. Membr. Sci*. **466**, 36-44 (2014).

24 Chen, P. C. & Xu, Z. K. Mineral-Coated Polymer Membranes with Superhydrophilicity and Underwater Superoleophobicity for Effective Oil/Water Separation. *Sci Rep* **3** (2013).

25 Solomon, B. R., Hyder, N. & Varanasi, K. K. Separating oil-water nanoemulsions using flux-enhanced hierarchical membranes. *Sci Rep* **4**, 5504 (2014).

26 Rajasekhar, T., Trinadh, M., Babu, P. V., Sainath, A. V. S. & Reddy, A. V. R. Oil-water emulsion separation using ultrafiltration membranes based on novel blends of poly(vinylidene fluoride) and amphiphilic tri-block copolymer containing carboxylic acid functional group. *J. Membr. Sci*. **481**, 82-93 (2015).

27 Chakrabarty, B., Ghoshal, A. K. & Purkait, M. K. Cross-flow ultrafiltration of stable oil-in-water emulsion using polysulfone membranes. *Chem. Eng. J*. **165**, 447-456 (2010).

28 Hu, L. *et al*. Photothermal-responsive single-walled carbon nanotube-based ultrathin membranes for on/off switchable separation of oil-in-water nanoemulsions. *ACS nano* **9**, 4835-4842 (2015).
